# Individual Variation in Cone Photoreceptor Density in House Sparrows: Implications for Between-Individual Differences in Visual Resolution and Chromatic Contrast

**DOI:** 10.1371/journal.pone.0111854

**Published:** 2014-11-05

**Authors:** Amanda L. Ensminger, Esteban Fernández-Juricic

**Affiliations:** Department of Biological Sciences, Purdue University, West Lafayette, Indiana, United States of America; University Zürich, Switzerland

## Abstract

Between-individual variation has been documented in a wide variety of taxa, especially for behavioral characteristics; however, intra-population variation in sensory systems has not received similar attention in wild animals. We measured a key trait of the visual system, the density of retinal cone photoreceptors, in a wild population of house sparrows (*Passer domesticus*). We tested whether individuals differed from each other in cone densities given within-individual variation across the retina and across eyes. We further tested whether the existing variation could lead to individual differences in two aspects of perception: visual resolution and chromatic contrast. We found consistent between-individual variation in the densities of all five types of avian cones, involved in chromatic and achromatic vision. Using perceptual modeling, we found that this degree of variation translated into significant between-individual differences in visual resolution and the chromatic contrast of a plumage signal that has been associated with mate choice and agonistic interactions. However, there was no evidence for a relationship between individual visual resolution and chromatic contrast. The implication is that some birds may have the sensory potential to perform “better” in certain visual tasks, but not necessarily in both resolution and contrast simultaneously. Overall, our findings (a) highlight the need to consider multiple individuals when characterizing sensory traits of a species, and (b) provide some mechanistic basis for between-individual variation in different behaviors (i.e., animal personalities) and for testing the predictions of several widely accepted hypotheses (e.g., honest signaling).

## Introduction

Evolution through natural selection needs intraspecific variation [1]. Thus, it is important to understand the nature and degree of phenotypic differences between individuals within populations [2]. Selection can maintain individual variation if phenotypes differ in fitness in heterogeneous environments [3], but even in homogeneous environments, individuals may specialize in traits to exploit different resources due to morphological or physiological specializations [4].

The visual system is one important means by which many organisms obtain information about their environment, so that they can make decisions about how to behave in a wide variety of contexts, including reproductive, foraging, and anti-predator situations [5]. Between-individual variation in visual signal detection and processing could potentially mediate variation in behavior. However, before establishing the link between perception and behavior, it is necessary to determine empirically if there is variation in sensory systems that that could lead to differences in visual perception.

Intraspecific variation in the receiver visual system can happen at different levels, such as between populations [6], morphotypes [7], and the sexes [8]. For example, bluefin killifish (*Lucania goodei*) living in springs differ from swamp populations in retinal characteristics in ways that match the properties of the light available in the different environments [6], [9], and ambient light conditions can affect mating and foraging behaviors [10]. There is also evidence for individual variation within sexes and ages in visual system properties in several vertebrate taxa, including humans [11], [12], non-human primates [13], [14], fish [9], [15], [16], birds [17], [18], and amphibians [19]. Despite this evidence, some trait estimates may show variation *across* individuals but if the variation is extensive *within*-individuals compared to *between*-individuals, then individuals may not be consistently different from one another. For instance, *within*-individuals, both absolute and relative cone densities vary between the right and the left eyes in European starlings (*Sturnus vulgaris*) [20], and between different parts of the retina in birds [21] and humans [22]. Therefore, it is important to assess whether the variation seen in visual traits is indeed consistently different between individuals given the within-individual variation across the retina and eyes. Between- and within-individual variances can be estimated with mixed models [2], [23], and the comparison of these two types of variation can be represented by repeatabilities [24], which indicate the degree to which individuals differ from each other compared to their own variation within- and across eyes. These approaches are increasingly used in studies of individual variation in behavior (methods reviewed in [25]), and should prove useful in testing for individual variation in visual traits as well (e.g. [26]).

In animal visual communication, the vast majority of research has been centered on variation between *senders* in the signal characteristics [27], but much of this literature assumes that signal detection and processing is the same across *receivers* [28], despite the evidence that receivers differ to some extent in visual properties, such as opsin expression, visual pigment sensitivities, and photoreceptor distributions [9], [16], [26]. In the context of visual signals, several factors could influence receivers' decisions, including luminance contrast, temporal resolution, chromatic contrast, and spatial resolution [29], [30]. For instance, the degree of chromatic contrast between color patches within the frill of the Australian frillneck lizards (*Chlamydosaurus kingii*) signals fighting ability [31], potentially influencing a receiver's decision to escalate or not agonistic interactions.

In birds, chromatic contrast (i.e., perceived degree of contrast between two surfaces based on chromatic cues) and spatial resolution (i.e., visual resolution, the ability to resolve two objects in the visual field as different) have been argued to influence the receiver's decisions in response to visual signals [30]. One visual trait that influences both chromatic contrast and visual resolution is the density of cone photoreceptors. The *relative* density of photoreceptors, among other factors, influences chromatic contrast because color discrimination is thought to occur through opponency mechanisms, which compare the photon capture of different types of cones [32]. Avian species, which are tetrachromatic, have four types of single cone photoreceptors which are implicated in, at least, chromatic vision: VS or UVS (violet sensitive or ultraviolet sensitive), SWS (short-wavelength sensitive), MWS (medium wavelength sensitive), and LWS (long-wavelength sensitive), as well as a fifth type of photoreceptor, the double cone, involved in achromatic/luminance vision and motion detection [33]. Avian single cones have been involved in three opponency mechanisms—LWS:MWS, SWS:UVS, [LWS+MWS]:SWS [34]. On the other hand, the *absolute* density of photoreceptors, among other important factors such as eye shape and size, influences visual resolution because the distance between two cones must be smaller than the retinal distance between two objects for an individual to resolve them [35].

We assessed the degree of between-individual variation in cone photoreceptor densities of house sparrows (*Passer domesticus*) taking into consideration within- and between- sources of individual variation. Additionally, we established the influence of a single retinal trait, cone densities, in modeled visual perception (visual resolution, chromatic contrast). We chose to focus on cone photoreceptors to assess levels of between-individual variation (rather than looking at optical components of the eye), as previous studies on birds have shown evidence of cone density variation between sexes [36] as well as between the right and left eyes [20]. We decided to use house sparrows because they exhibit sexual dimorphism and display visual signals, including a wingbar [37], during mating and aggressive interactions. The contrast of the wingbar against the surrounding wing coverts has been documented as a dominance signal, and linked to female preferences [38]. The wingbar may also be an honest indicator of some aspects of condition, as parasitic chewing lice preferentially create holes in the white wingbar [39], [40]. Therefore, in this study we modeled the perception of the wingbar.

We first tested whether absolute cone densities varied between sexes, eyes, and individuals, using a linear mixed model approach [41], which is more powerful statistically than the coefficient of variation reported in previous studies because these models can test whether the between-individual variation outweighs within-individual variation (e.g. [11]). Second, we explored the relationships between the absolute densities of the different cones types to test whether the primary difference between individuals was in total cone densities but the relative representation of cones was similar across individuals (i.e., positive relationships between cone type densities), or whether the primary difference between individuals was in the relative representation of cones while total cone densities were similar (i.e., negative relationships between cone type densities) across individuals. Third, we tested whether individuals differed in cone type proportions, as they are important to chromatic contrast. Fourth, we assessed the between-individual variation in modeled chromatic contrast and visual resolution. Finally, we explored the relationship between individual estimates of chromatic contrast and visual resolution to establish whether high performance in one perceptual component would be associated with high or poor performance in the other, or whether the two perceptual components would not be linked.

## Materials and Methods

We used 13 male and 13 female adult house sparrows (*Passer domesticus*) in this study.

### Ethics Statement

Birds were caught in mist-nets and potter traps in Tippecanoe County, Indiana, USA, in four residential areas, with GPS coordinates: 1) 40.384607, −86.862510, 2) 40.459135, −86.905086, 3) 40.407410, −86.883082, and 4) 40.372615, −86.872707. We obtained verbal permission from the residents to trap in these locations. This study did not involve endangered or protected species. Permission to capture house sparrows in Tippecanoe County was granted by the Indiana Department of Natural Resources, under an Indiana Scientific Purposes License to A.L.E., License number 12-0083. As house sparrows are not a protected species, no specific Federal license was required. All research activities were approved by the Purdue University Animal Care and Use Committee (protocol 1111000242).

### Animal housing

Animals were housed at Purdue University animal facilities in 0.61×0.61×0.76 m cages on a 14 hr. light/10 hr. dark cycle. Birds were held between zero (same day as capture) and 40 days before data collection, with an average of 9 days ±2.16 (SEM); the number of days in captivity did not significantly affect cone densities (P>0.1). They were all fed the same diet of *ad libitum* white millet, which contains enough carotenoids to sustain retinal carotenoid levels for at least eight weeks [42]. Water was available at all times.

### Retinal procedures

Fifty-two retinas were used in this study (two per bird, 26 birds). Immediately after euthanasia by CO_2_-asphyxiation, we measured eye axial length, transverse length, and corneal diameter (in mm), and hemisected the eye just posterior to the lens at the ora serrata using a razor blade. Vitreous humour was removed using tweezers and spring scissors under a dissecting microscope, after which the eyecup was saturated in phosphate buffered saline solution (PBS, pH 7.2–7.4). We removed the retina by detaching the choroidal layer from the sclera and severing the optic nerve. We did not attempt to remove any pigmented epithelium that did not spontaneously detach with the choroid to avoid damaging the photoreceptor layer and biasing our counts. We then placed the retina in 4% paraformaldehyde for 30 min to strengthen the retina for further manipulation, so that oil droplets were not lost from the retina and the cone matrix was kept in-tact [21]. The retina was flattened with the vitread side up on a slide by making several radial cuts (six to eight) with spring scissors, and gently unrolling the edges with small paint brushes. We applied two drops of PBS to the retina, flipped the cover slip over with the retina attached so that the sclerad side was up, coverslipped it, and sealed it with glue.

We viewed the retinas with an Olympus BX51 microscope. We used the SRS (Systematic Random Sampling) Image Series Acquire workflow module of Stereo Investigator v.10 (MBF Bioscience). We used this workflow to (1) trace the perimeter of the retina, the pecten, and to trace out (eliminate) any areas with pigmented epithelium obscuring the retina, with a 4× objective and 0.1 numerical aperture, (2) fit a systematic random grid over the traced retina, with the following specified stereological parameters [43]: 280 squares, number of sections  = 1, ssf (stereological sampling fraction)  = 1 per retina, thickness  = 1, tsf (thickness of sampling fraction)  = 1 per retina, and (3) photograph each square using a 40× objective lens with a numerical aperture of 0.1, under both bright field and epifluorescent illumination. The counting frame (50 µm ×50 µm; 0.0025 mm^2^) was positioned in the upper left corner of all squares.

**Figure 1:**
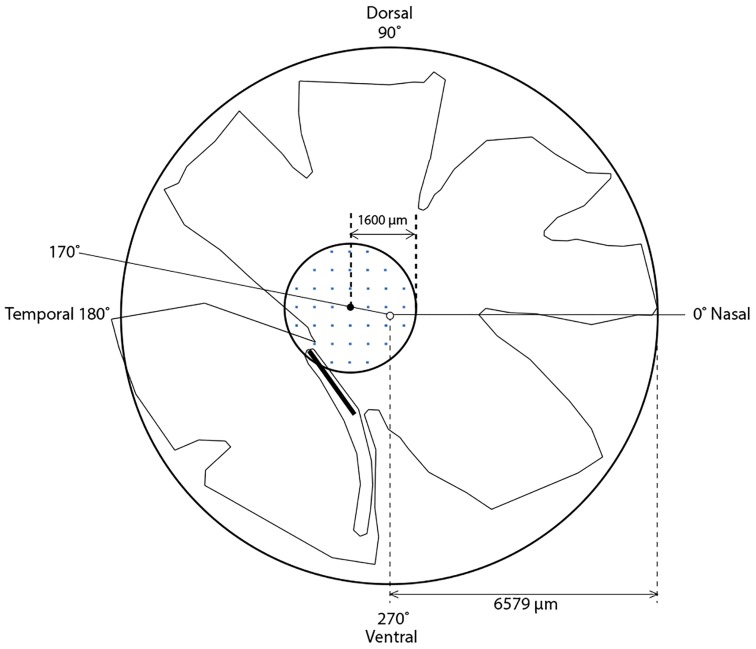
Schematic representation of a representative house sparrow right eye retina, indicating the fovea (small filled black circle), the center of the retina (small open circle), the pecten (thick black line); for details on the estimation of the locations of these retinal features, see Appendix S1. The approximate perifoveal area sampled in our study (1600 µm radius around the fovea), is indicated by the bold circle centered on the fovea; the area of this representative retina is approximately 88.96 mm^2^, and the perifoveal region (8.04 mm^2^) covers 9% of this retina. Small blue squares inside the perifoveal region hypothetically represent the 0.0025 mm^2^ counting frame sites (not to scale) determined by systematic random sampling.

We chose to concentrate our cell counting efforts on the perifoveal area for two important reasons. First, a previous study in humans found the highest levels of between-individual variation in photoreceptor densities around the fovea [11]. Second, the fovea has been implicated in birds as the center of chromatic and achromatic visual resolution [36], [44], thereby potentially having a central role in visual attention [45]. After capturing the images, we manually determined the location of the fovea on each retina using the tip and angle of the pecten as a landmark (see Appendix S1). We had previously determined the location of the house sparrow fovea as being on average 1,404 µm from the pecten tip, at a 98° angle from the pecten (see Appendix S1). For the analyses presented here, we chose to count cells in sites that lay within a 1,600 µm (23.3°) radius perifoveal region (Fig. 1; Appendix S1); this region, being 8.04 mm^2^ in area, covers approximately 9% of the total area of the retina (Fig. 1). We were not able to determine the location of the fovea at the time we took the pictures with Stereo Investigator, because the fovea cannot be seen in a non-stained wholemounted retina and we were time-limited to take pictures on the fresh retina to avoid oil droplet degradation, which can begin to occur after three hours of microscope use. Therefore, during microscope use, we fit the grid to the entire retina, and later determined the location of the perifoveal region containing sites that we would then count. Our methods could have generated a bias in the density estimates, which we minimized by (a) including eccentricity (distance away from the foveal region) as a covariate to account for the variation in distance between sites, and (b) incorporating a random intercept that accounted for the differences between individuals in the number of sampling replicates.

A total of 1,386 sites were included in the initial sampling area across the 52 retinas. However, 326 of these sites were not able to be counted; we only counted sites where (1) all cone types were represented, and arranged in a matrix-like pattern [46], (2) no pigmented epithelium or retinal debris was blocking any part of the image, and (3) no part of the image was blurry (Appendix S1). When part of the image did not meet these criteria, we divided the counting frame into four quadrants, counted the quadrants that met these criteria, and later divided by the area sampled to calculate density. After eliminating sites that did not meet these criteria, we counted a total of 1,060 sites (525 were from the left eye, and 535 from the right eye), with an average of 20±0.65 (SEM) sites per retina. Retinas did not differ significantly from random expectations in the number of sites counted (Chi-square test: χ^2^_51_ = 55.5, p = 0.31). On average, the number of sites counted per individual was 41 (min 29, max 54). For the average retina, the summed area counted constituted about 0.8% of the perifoveal region. Stereological estimates can be found in Appendix S1.

In birds, each cone type is associated with a specific type of oil droplet, which is an organelle thought to enhance the discrimination ability of the visual pigments [47]. There are five types of oil droplets: T-type (associated with the UVS single cone), C-type (associated with the SWS single cone), Y-type (associated with the MWS single cone), R-type (associated with the LWS single cone), and P-type (associated with the principal member of the double cone). Oil droplets differ in size and in color because of the differences in carotenoid types and their concentrations [21], [47]. Therefore, cone types can be identified by their oil droplets, and oil droplet densities have been used to estimate the density of each type of cone [21]. We used criteria given by Hart [21] to identify oil droplets, based on color, size, and plane in the retina (Appendix S1); we have used these criteria successfully before (e.g., [48]). Oil droplets were counted using ImageJ (http://rsbweb.nih.gov/ij/). We did not attempt to calculate the total population size of cones across the entire perifoveal region. Instead, we calculated the density of cones at each sampled site, as the number of cells counted divided by the area counted at that site. For example, a site that had 163 cones and a counting area of 0.0025 mm^2^ would have a density of 65,200 cells/mm^2^.

### Chromatic contrast

We used the high-light photon catch photoreceptor noise model of Vorobyev and Osorio [49] to estimate chromatic contrast, which is a measure of the distance between the quantum catch of two objects in the avian tetrahedral color space (Appendix S2). Chromatic contrast is measured in units of Just Noticeable Differences (JNDs), where lower values indicate less degree of visual contrast between two objects based on the visual physiology of the species. We estimated chromatic contrasts for the 52 retinas (26 individuals, with each eye estimated separately). All parameters in the model were the same for each individual except their cone densities.

We estimated the chromatic contrasts of the male house sparrow's wingbar against the lesser and greater wing coverts. We chose this plumage trait because it is a signal used in both mate choice and agonistic interactions [38], [50], and we chose to contrast the wingbar with the surrounding plumage in the models because the way in which house sparrows display the wingbar appears to enhance the visual prominence of these signals in relation to the surrounding plumage [37], [50]. We measured the reflectance of the plumage of six live male house sparrows with a StellarNet EPP2000 portable spectroradiometer (StellarNet-Inc., FL); we used six males in order to determine if between-individual differences in chromatic contrast would exist despite variation among the signal properties. The fiber optic cable was held at 45° from the feather surface, with the light shining in the direction of the distal end of the feathers. The white standard was the RS50 provided by StellarNet (StellarNet-Inc., FL), which is a 50 mm diameter white reflectance standard made of Halon, and reflects >97% of the light from 300–1700 nm. We measured the dark reference by holding the probe against the white standard with all light sources off. From 300–700 nm, ten measurements were taken per plumage region, per bird, and averaged across measures at each wavelength to produce an average spectrum for each region (wingbar, greater coverts, and lesser coverts). The deuterium lamp produces an artifactual spike between 650 and 655 nm, which we manually smoothed for each measurement before averaging the spectra.

We calculated chromatic contrast, for each of the 26 house sparrows for which we measured cone densities, using Avicol v6 ([51]; Vorobyev and Osorio's tetrachromatic model [49]; Appendix S2). To calculate chromatic contrast, we used the following parameters: (1) reflectance of the wingbar, (2) reflectance of the plumage surrounding the wingbar (greater and lesser coverts), (3) ambient light irradiance (described in Appendix S2), (4) relative densities of cone photoreceptors (data presented in the main text), and (5) spectral sensitivities of each single-cone type (described in Appendix S2).

### Visual resolution

We used anatomical spatial resolving power as an estimate of visual resolution. Our study aim was to test whether the existing degree of between-individual variation in cone densities would translate into significant between-individual variation in visual resolution. To isolate the effect of cone densities, we assumed that other traits (e.g., shape of the eye, refractive indices of cornea and lens, etc.) that can influence visual resolution were the same across individuals. Therefore, our estimates of spatial resolving power should be considered as relative between birds rather than absolute. Nevertheless, we followed the standard calculations to estimate spatial resolving power considering eye size and cone densities [52]–[56].

Resolving power was estimated from eye axial length and cone densities for each sampling site, following Tamura & Wisby [57]. The minimum separable angle (α) between adjacent cones was calculated as: 
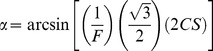



*F*, focal length, was calculated as 0.6× *eye axial length* [56]. The value 0.6 has been derived specifically for birds [58], [59], and is used as an accepted parameter in estimations of focal length for diurnal birds [60], like the house sparrow. We did not have eye axial length measures for 18 of the 52 retinas due to issues during extraction and measurement with the digital caliper. Since there was relatively low variation in axial length measurements (n = 34, CV  = 0.04), we used the average axial length (6.75 mm) for retinas that were missing that value. We also ran additional models to support this method (Appendix S3). We used the conversion factor, 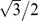, to incorporate a hexagonal photoreceptor matrix [35]. The cone separation distance (*CS*) was calculated as 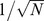, where *N* is the total density of cones (cells/mm^2^), including all four single cone types and the double cones. The value 2*CS* is twice the cone separation distance, assuming that the minimum separable angle is subtended on the retina by one period of the grating. We report the average spatial resolving power for each bird (averaged across sites and eyes). We also report the range of variation in spatial resolving power calculated from the site with the highest cone density. This is because studies on spatial resolving power base their calculations on peak retinal ganglion cell densities across the retina (e.g. [61]).

### Statistical analyses

All statistical tests were performed in SAS 9.3 [62]. All residuals were approximately normal. Throughout, we present means and standard errors.

#### Cone densities and cone type proportions

To test for individual variation in cone densities and cone type proportions, we analyzed each type of cone in a separate general linear mixed model [23]; we also analyzed total cone densities (all cone types together) as well as all single cones together. We treated each site on the retina as a data point (N = 1,060). To analyze absolute cone densities, the response variable was the cone density at that site (the cells counted at that site divided by the area counted in mm^2^). To analyze cone type proportions, the response variable was the number of counted cells of a given type of cone, at that site, divided by the total number of cones at that site (both single and double); the residual proportions were approximately normal, and so we did not transform them (Appendix S4). In each model, we included the following fixed effects: sex, eye (left or right), eccentricity (distance from the fovea, since cone densities are known to decrease with eccentricity), the interaction between eccentricity and eye, the observer who counted the site, and the date of retina extraction (to control for unintended methodological changes). We also ran models including eye axial length in the subset of data for which we had axial length values from each individual (34 retinas), but axial length did not significantly affect absolute cone densities for any cone type (p-value from 0.31 to 0.72). Hence, we excluded this parameter from the models in order to use the full data set.

Also in each of the aforementioned models, we included three random terms in order to test for between-individual variation in cone densities and cone type proportions: (1) between-individual variance across left eyes, (2) between-individual variance across right eyes, and (3) the covariance across eyes. We also partitioned the residual variance into left and right eyes, which represents the within-retina variation, so that we could calculate adjusted repeatabilities [24] for the left eye and the right eyes, separately. For example, to calculate the adjusted repeatability for the left eyes, we divided the between-individual variance estimate of the left eyes by the sum of that variance and the residual variance estimate (within-retina variance) for the left eyes. This repeatability represents the degree to which individuals' left eyes differ from each other. Repeatablities for the right eyes were then calculated separately in the same way. To quantify the degree to which individuals were consistently different across their two retinas, we estimated the covariance correlation, which was calculated as the covariance divided by the square-root of the product of the variances [41]. We tested the significance of each of these three random terms with Likelihood Ratio Tests [63]. A significant test for either eye would indicate that individuals differ consistently from each other despite the within-individual variation across sites at the retina level, while a significant covariance would indicate that individuals differ consistently from each other despite the within-individual variation across their own two retinas.

We used Pearson correlations to test for linear relationships between each pair of cone types. The data points (N = 26 for each pairwise correlation) were the mean density of cells (cells/mm^2^) for each bird (averaged across sites and eyes).

#### Chromatic contrasts and visual resolution

We analyzed chromatic contrasts using general linear mixed models. Each data point was the chromatic contrast estimate (JNDs) for a retina-wingbar combination (52 retinas ×6 wingbars: N = 312). We included the following fixed effects: the identity of the wingbar, sex, and eye. Individual was included as a random intercept term. We also analyzed visual resolution using general linear mixed models, where each data point was the spatial resolving power (cycles/°) calculated for each site on the retina (N = 1,060). The fixed effects in the model were: sex, eye, eccentricity, eccentricity × eye, and observer who counted the site. Individual was also included as a random intercept term.

For both chromatic contrast and visual resolution, we calculated the adjusted repeatability to estimate the degree of between-individual variance as the variance of the random individual intercept divided by the sum of that variance plus the residual (within-individual) variance. We tested the significance of the between-individual variance with a Likelihood Ratio Test.

We used Pearson correlations to test whether chromatic contrast and visual resolution were related. We ran six correlation tests, one for each wingbar; the data points in each correlation (N = 26) were the chromatic contrast averaged across eyes for each bird and the average visual resolution for each bird (averaged across sites and eyes).

## Results

### Absolute cone densities

Table 1 contains summary statistics on absolute cone densities within the perifoveal region for the 52 retinas (summary statistics per retina can be found in Appendix S5). Absolute cone densities decreased with distance from the fovea (eccentricity) for total cell densities, on average by 13±2 cells/mm^2^ for every µm from the fovea (F_1, 1008_ = 105, P<0.001); this relationship was not significantly different between left and right eyes (P>0.1; Appendix S6). We did not find significant differences in cone densities for any type of cone between males and females (P>0.1; Appendix S6) or between the right and left eyes (P>0.1; Appendix S6). There was also no evidence of a relationship between cone densities and eye size (P>0.1 for axial length, transverse diameter, and corneal diameter, Appendix S6). Details on all fixed effects included in the models of absolute cone densities can be found in Appendix S6.

**Table 1:** Average, minimum, and maximum densities (cones/mm^2^) of the 26 house sparrows.

Cone type	Mean Density*	Percentage	Minimum bird mean	Maximum bird mean	Minimum site	Maximum site
All cones	71,034±1,288	100.00%	60,353	82,029	24,800	155,600
Double	26,243±472	36.94%	21,929	32,757	8,000	64,400
Single	44,791±837	63.06%	35,701	56,390	14,000	91,200
LWS	11,812±374	16.63%	9,067	18,311	2,000	25,067
MWS	13,587±285	19.13%	10,476	16,589	4,000	25,600
SWS	13,508±250	19.02%	10,481	16,000	3,600	31,600
UVS	5,884±147	8.28%	4,529	7,280	800	18,000

*Mean density: average of bird means ± SEM; values are for pooled retinas for each individual.

**Figure 2:**
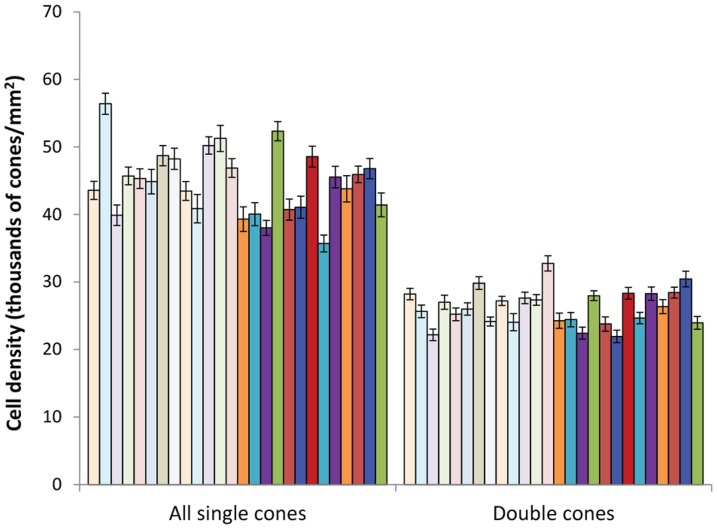
Between-individual variation in absolute densities of double and single cone photoreceptor densities. Each bar represents the mean and standard error for an individual. Light bars are females (n = 13), dark bars are males (n = 13).

These mixed models revealed that individuals differed significantly in the absolute density of cones (Fig. 2; Table 2). This was true for right and left eyes separately, suggesting that individuals were consistently different from one another within each eye, given the variation among sites. The significant positive covariance across eyes suggests that the differences between individuals in each eye were maintained across their eyes (Table 2). The individual with the highest density of all cones had 36% higher density than the individual with the lowest density (Table 1). The absolute density of the LWS cones varied the most (100% from the individual with the lowest to that with the highest density, Appendix S5), but also had the highest repeatabilities (0.38) and covariance correlation (0.74), indicating a high variability in this cone type across individuals, but a low variability in absolute densities within-individuals.

**Table 2:** Repeatabilities and Likelihood Ratio Tests of individual variation in photoreceptor densities, for each eye, and their covariance.

	Left eye				Right eye				Covariance			
Cone type	r_a_	d2RLL	df	p	r_a_	d2RLL	df	p	r_c_	d2RLL	df	p
All cones	0.24	78.96	2	<0.001	0.17	58.94	2	<0.001	0.47	3.95	1	0.047
Double	0.25	100.9	2	<0.001	0.18	62.49	2	<0.001	0.52	5.19	1	0.023
Single	0.25	81.2	2	<0.001	0.19	72.32	2	<0.001	0.57	6.31	1	0.012
UVS	0.21	77.41	2	<0.001	0.17	60.88	2	<0.001	0.66	8.52	1	0.004
SWS	0.20	68.3	2	<0.001	0.15	46.23	2	<0.001	0.61	6.52	1	0.011
MWS	0.21	67.81	2	<0.001	0.16	53.57	2	<0.001	0.59	6.13	1	0.013
LWS	0.38	160.71	2	<0.001	0.38	202.74	2	<0.001	0.74	15.61	1	<0.001

r_a_: Adjusted repeatabilities [24]; r_c_: Covariance Correlation [41]; d2RLL  =  the difference between the -2*restricted log likelihoods of models with and without the variance component, used in a Likelihood ratio test as a chi-square statistic [41].

### Absolute density relationships between cone types

**Figure 3:**
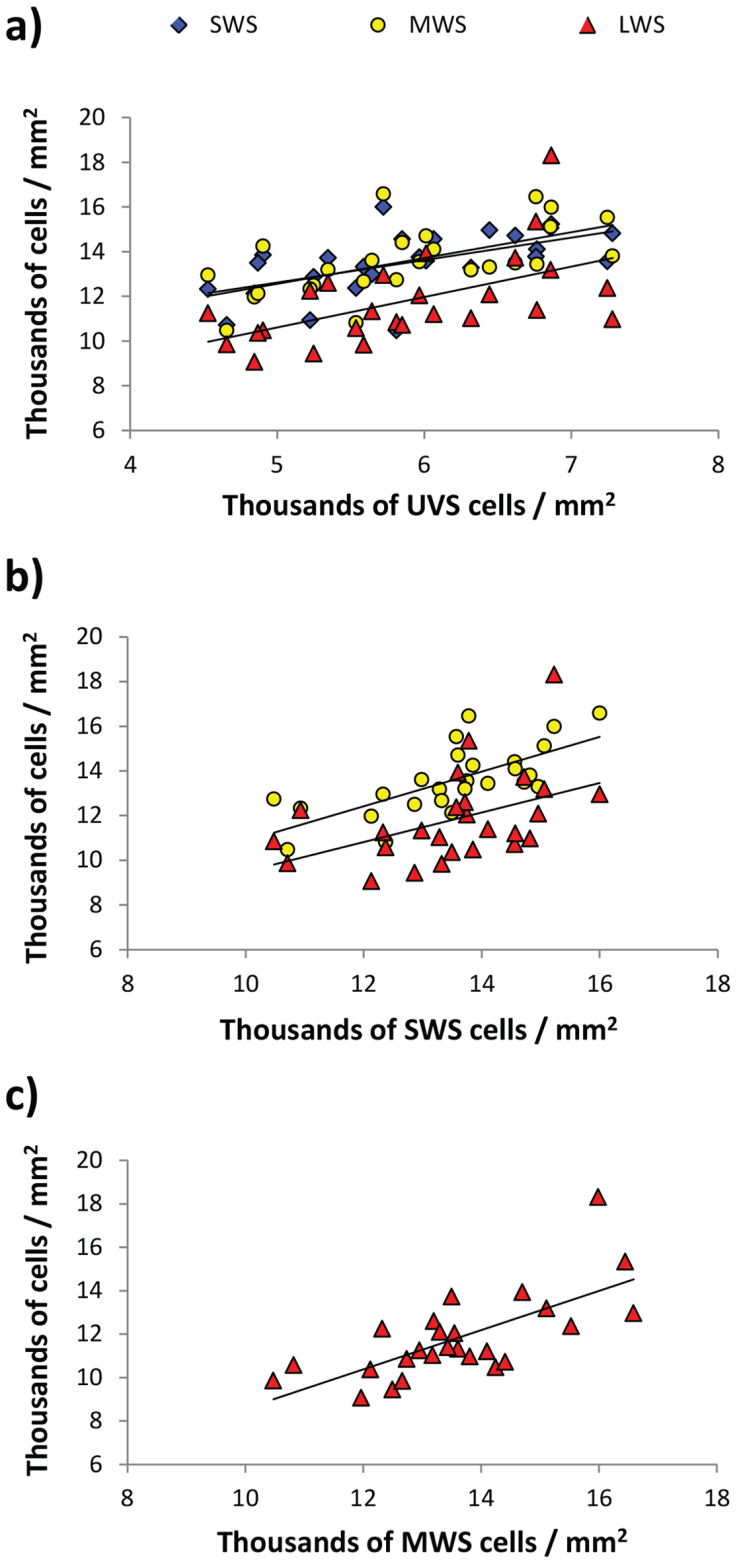
Correlations between densities of the single cones. Each data point is the average density (thousands of cells/mm^2^) for an individual (pooled eyes) for that type of cone. (a) LWS (red triangles), MWS (yellow circles), and SWS (blue diamonds) versus UVS densities; (b) MWS and LWS versus SWS densities; (c) LWS versus MWS densities. Pearson correlations, n = 26; LWS-MWS r = 0.70, *P*<0.001; LWS-SWS r = 0.46, *P* = 0.02; LWS-UVS r = 0.55, *P* = 0.003; MWS-SWS r = 0.71, *P*<0.001; MWS-UVS r = 0.61, *P* = 0.001; SWS-UVS r = 0.58, *P* = 0.002.

The absolute density of all single cones was positively correlated with the absolute density of double cones (Pearson correlation, *r* = 0.56, *P* = 0.003, n = 26), and all pair-wise relationships of the four single cone types were positive and significant (Fig. 3). This suggests that some individuals packed more of all cone types in their retinas than others.

### Between-individual variation in cone type proportions

**Figure 4:**
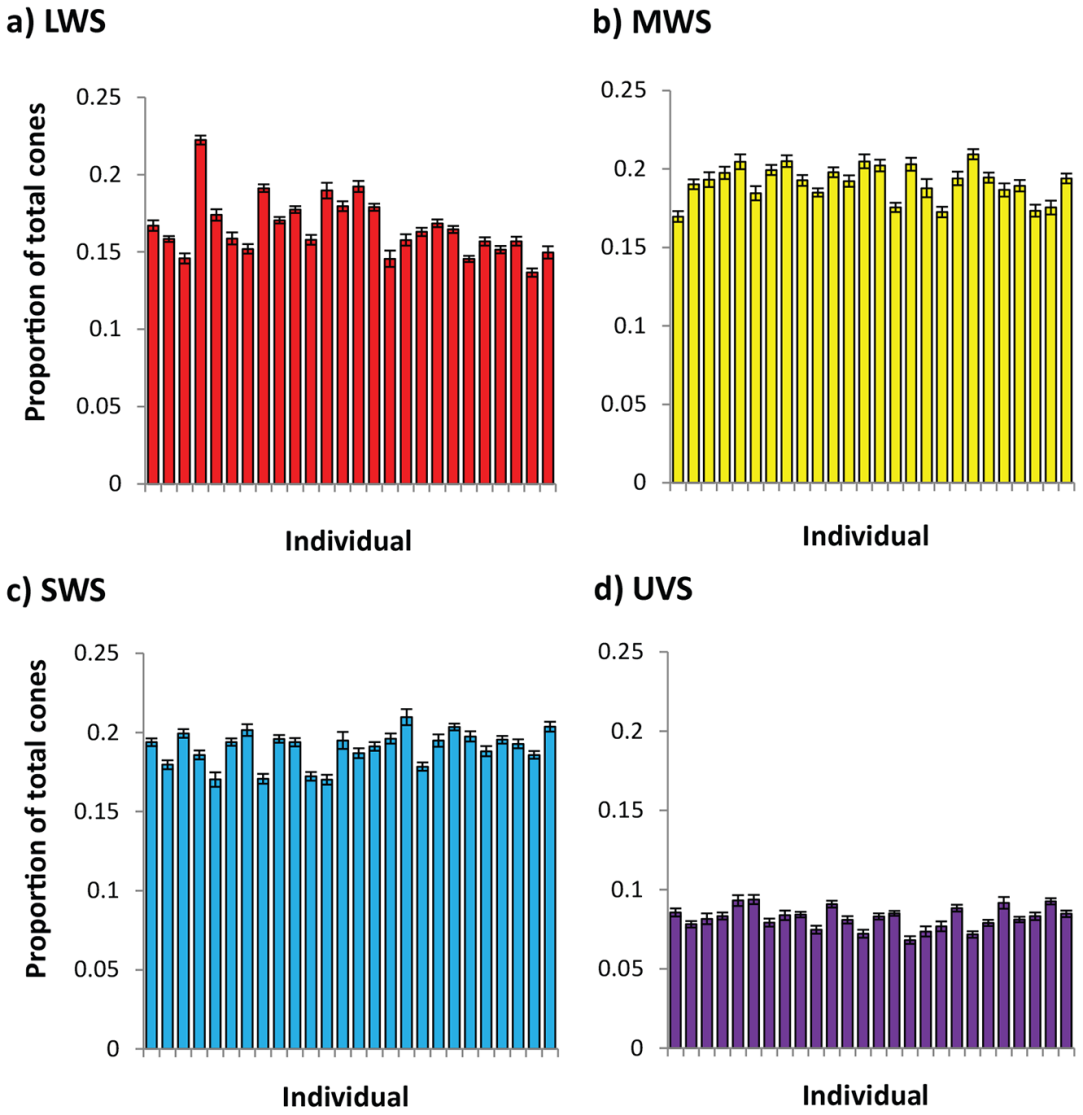
Between-individual variation in single cone type proportions out of the total density of cones (including double cones—not shown—which make up on average 37% of all cones). Each bar represents the mean and standard error for an individual—the errors represent within-individual variation across sites and eyes.

We found strong evidence of consistent between-individual variation in cone type proportions, for double cones and for each type of single cone (Fig. 4, Table 3). Adjusted repeatabilities within each eye were highest for LWS single cones and the double cones (Table 3). All covariances between the eyes were substantial, with UVS cones having the lowest covariance correlation, and LWS having the highest (Table 3). These results suggest that individuals differ consistently from each other in the proportions of each cone type.

**Table 3:** Repeatabilities and Likelihood Ratio Tests of individual variation in cone proportions, for the left eye and right eye, and their covariance.

	Left eye				Right eye				Covariance			
Cone type	r_a_	d2RLL	df	p	r_a_	d2RLL	df	p	r_c_	d2RLL	df	p
Double	0.30	141.6	2	<0.001	0.29	135.2	2	<0.001	0.88	22.7	1	<0.001
UVS	0.20	70.44	2	<0.001	0.19	66.45	2	<0.001	0.66	8.91	1	0.003
SWS	0.26	91.95	2	<0.001	0.27	107.1	2	<0.001	0.79	15.5	1	<0.001
MWS	0.17	63.95	2	<0.001	0.15	54.72	2	<0.001	0.78	11.9	1	<0.001
LWS	0.47	276.4	2	<0.001	0.50	319.4	2	<0.001	0.97	45.9	1	<0.001

r_a_: Adjusted repeatabilities [24]; r_c_: Covariance Correlation calculated according to [41]; d2RLL  =  the difference between the -2*restricted log likelihoods of models with and without the variance component, used in a Likelihood ratio test as a chi-square statistic [41].

### Between-individual variation in chromatic contrast

**Figure 5:**
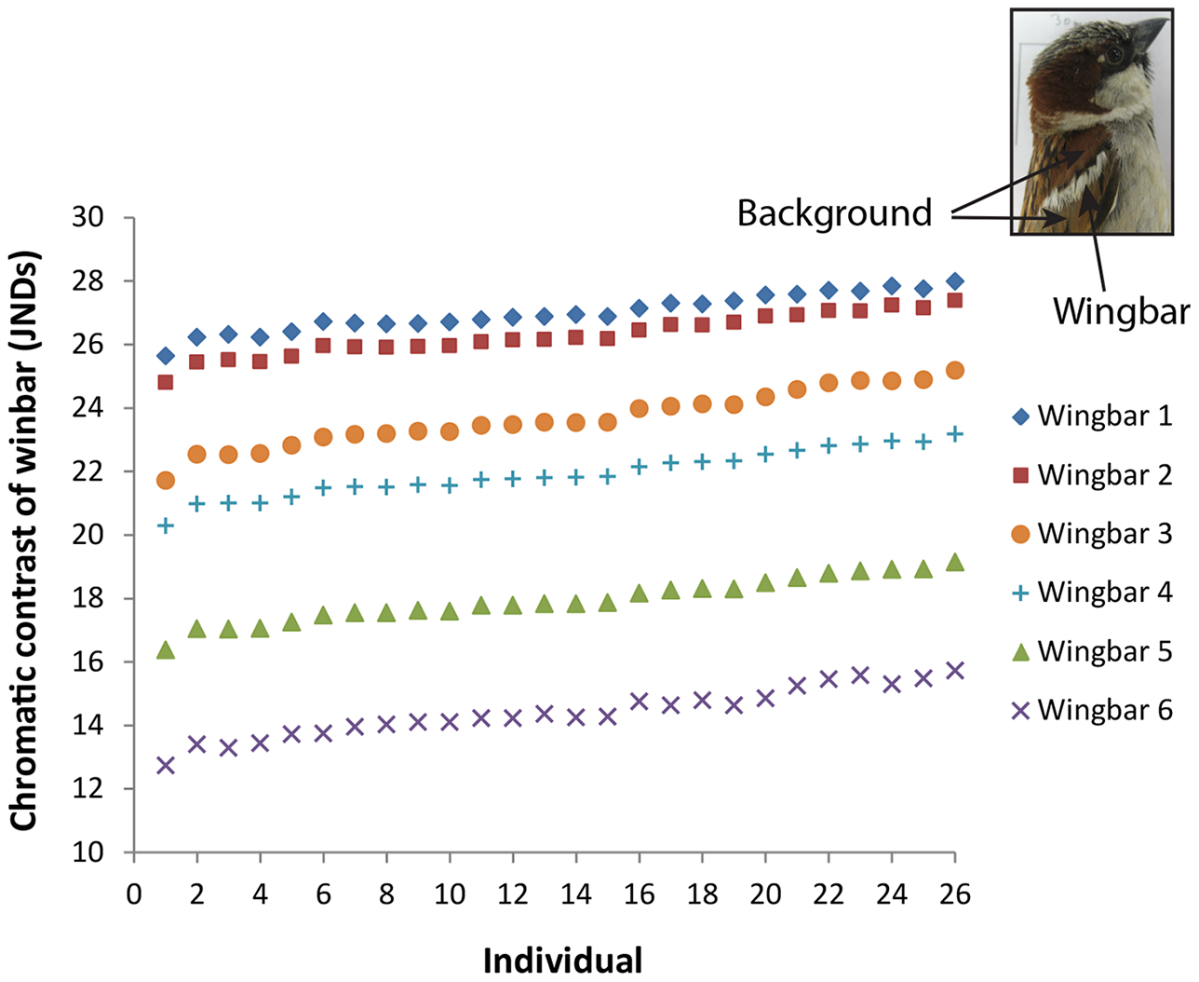
Individual variation in estimated chromatic contrast of 26 individual house sparrows viewing the six wingbars against the surrounding wing coverts. Each data point is the mean for an individual. Error bars are not shown because they mostly did not extend past the data points. Individuals (x-axis) are ordered according to their chromatic contrasts for wingbar 1 (individual 1 had the lowest contrast, individual 26 had the highest contrast).

From the perspective of the viewer, the six different wingbars varied by 87% in their average chromatic contrasts: from 14.40±0.15 JNDs to 26.99±0.02 JNDs. Wingbar identity had a significant effect on chromatic contrast across all 26 birds (F_5,280_ = 4297.6, *P*<0.0001). Sex was not significant for wingbar contrasts (F_1,24_ = 0.03, *P* = 0.87). We also did not find a significant effect of eye (left versus right) on contrasts of the wingbar (F_1,280_ = 0.19, *P* = 0.66). Regardless of the specific wingbar, the 26 individuals differed significantly in chromatic contrast estimates of the wingbar (Fig. 5; repeatability  = 0.64, Likelihood Ratio Test: χ^2^_1_ = 224.71, *P*<0.001). The maximum range in chromatic contrast across individuals for a particular wingbar was 3.5 JNDs (16%, wingbar 3 in Fig. 5). This within-wingbar variation was 28% of the range between wingbars (12.6 JNDs, from the average value of wingbar 6 to the average of wingbar 1). These results suggest that, for any given wingbar, individuals are expected to differ in the degree of chromatic contrast they perceive.

### Between-individual variation in visual resolution

**Figure 6:**
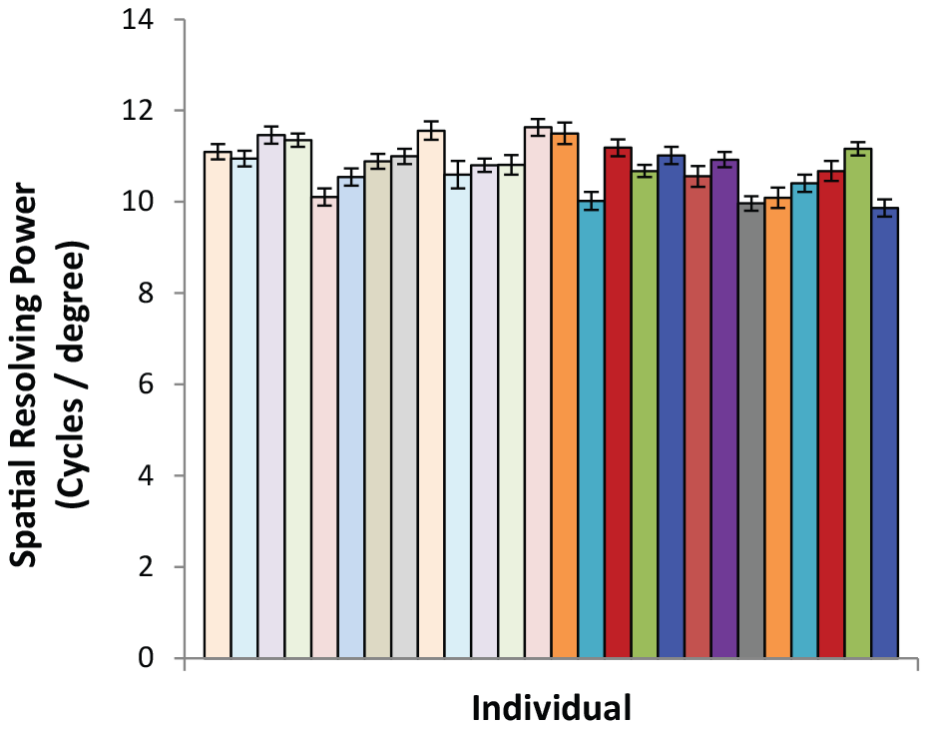
Visual resolution as calculated from cone densities for each of the 26 individuals. Light-filled bars are female birds (n = 13), and dark-filled bars are male birds (n = 13). Bars show the means and SE for each individual.

We found that anatomical spatial resolving power (a proxy of visual resolution) varied significantly between individuals (Fig. 6; repeatability  = 0.15, Likelihood Ratio Test: χ^2^_1_ = 98, *P*<0.001), after controlling for the significant effects of eccentricity (partial β = −0.001±0.0001, *P*<0.001; Appendix S3). The average anatomical spatial resolving power varied across bird by 18% (from 9.87 to 11.63 cycles per degree), with a mean of 10.8 (±0.04) cycles per degree (Fig. 6). The peak anatomical spatial resolving power varied across bird by 35% (from 11.67 to 15.81 cycles per degree), with a mean peak of 13.30 (±0.18) cycles per degree. These values show the potential variation among individuals relative to each other, but do not reflect absolute estimates of spatial resolving power, as other components (e.g., eye shape, refractive index of cornea and lens) were not measured. The significant between-individual variation suggests that a single retinal trait, cone densities, can potentially result in some individuals being better able to resolve visual stimuli than others.

### Relationship between chromatic contrast and visual resolution

**Figure 7:**
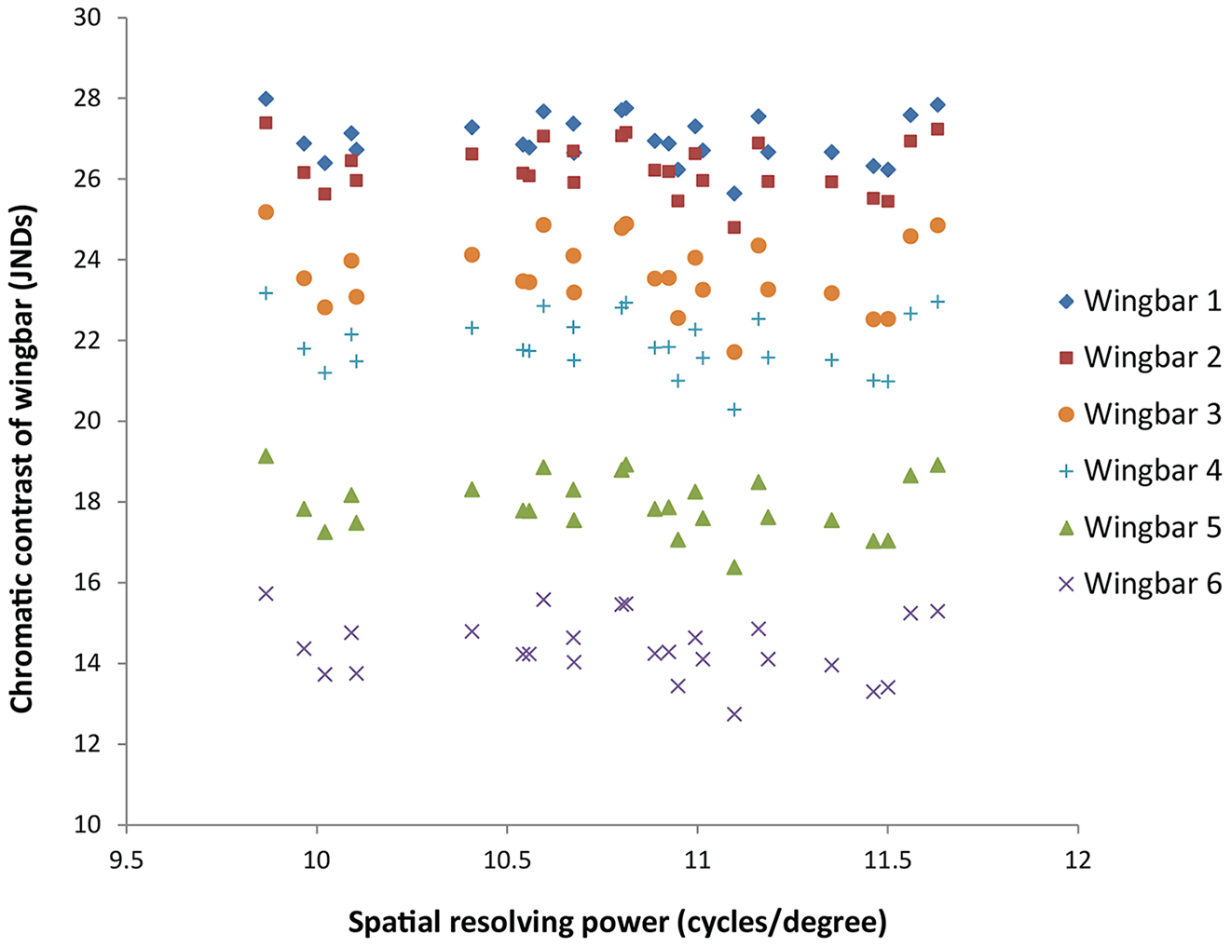
Chromatic contrast of the six wingbars versus spatial resolving power for 26 house sparrows. Each data point is the spatial resolving power and the average chromatic contrast for that individual, modeled for that wingbar.

We did not find a significant correlation across individuals between their modeled perception of chromatic contrast and their anatomical spatial resolving power, either in a positive or a negative direction. This was true for models of all six wingbars (Fig. 7; Pearson *r* varied between 0.12 and 0.20, *P*>0.1 for all). This suggests that individuals would have neither high performance in both visual tasks simultaneously, nor high performance in one component over the other.

## Discussion

Our results show that there is significant between-individual variation in a key trait of the visual system—the density of cone photoreceptors—in a wild population of birds. House sparrows differed in the absolute density of both single cones, involved in chromatic vision, and double cones, involved in achromatic/motion vision in an area of the retina (perifoveal) that has been associated with the center of visual attention [64]. Interestingly, the between-individual variation in cone densities of house sparrows (cumulative CV = 0.13) is actually comparable to that reported in humans (cumulative CV = 0.10; [11]) considering retinal areas around the fovea (within 7° of the fovea).

We also found through modeling approaches that cone density differences could translate into between-individual variation in two important visual processes likely involved in social interactions in house sparrows: chromatic contrast and visual resolution. We found that birds ranged in peak resolving power by 4.14 cycles per degree (35%). This is a moderate range when compared to previous studies showing intraspecific variation in spatial resolving power of birds based on peak retinal ganglion cell densities, including five species of cockatoos (3.2% to 16.7%) [65], budgerigars (23%), Bourke's parrots (46%), chickens (54%), and pigeons (91%) (data and references in [61]). However, our results should be taken with care for two main reasons. First, we did not measure all the factors that could be affecting visual resolution, such as, eye shape, the refractive indices of the lens and cornea, or the density of retinal ganglion cells [61]. Nevertheless, the between individual variation in cone densities remained significant even after controlling for eye size. We also measured corneal diameter and transverse eye length on each individual, but they did not have significant effects on the reported variation in cone densities (Appendix S6). Second, individuals with low cone densities (hence, lower visual resolution according to our results) could have compensated with variations in some of the factors that were not measured, resulting in no net differences in perceived visual resolution between individuals. Future behavioral studies would be necessary to determine whether the predicted differences in perception translate to behaviors that are ecologically relevant (e.g., foraging, mate choice, etc.).

Cone densities varied between individuals irrespective of sex and eye. This density variation could be the result of changes in two parameters. First, the area of the retina may differ between individuals [66], but the total number of cones may remain the same. Second, the area of the retina may be similar across individuals, but the total number of cones may differ. Our findings support the latter scenario (i.e., some individuals pack cones more tightly than others) as eye axial length did not show much variation among the birds in this study (CV = 0.04), and between-individual differences in cone densities were independent of eye size.

Cone density estimates at different sites on the retina are complicated by the fact that cell densities decreased with distance from the fovea. This heterogeneity of cell densities across the retina can create the appearance of individual differences in two ways. First, if the location of density estimates differed between individuals, cell densities would be higher for those individuals sampled closer to the fovea. Our sampling scheme measured cell densities at many randomly-chosen sites per individual, but the exact location of those sites varied in distance from the fovea for the different individuals due to the systematic random sampling stereological procedure; this could have led to finding significant between-individual differences in absolute cone densities. However, the change in cell densities per unit distance from the fovea was small within the sampled region (−13±2 cells/mm^2^ per µm eccentricity from the fovea), suggesting that the region sampled was relatively homogeneous, and our random intercepts accurately represent differences in absolute cell densities. Second, individuals may have differed in the rate of change in cell densities per unit distance from the fovea. An analysis of the interaction between individual and eccentricity in a GLM suggested that individuals did not significantly differ in the rate of change in cell densities (F_25,965_ = 0.77, P = 0.79). Therefore, while it is important to consider these sources of error, our results do suggest that individuals differ in their absolute cone densities.

There are multiple factors that could explain the between-individual variation in cone densities, including genetic variation, age, condition, and/or developmental conditions. Genetic variation in humans has been shown to disrupt cone mosaics, especially in the region around the fovea [67]. The retina goes through changes over time. In humans, loss of photoreceptors occurs during macular degeneration with age [68], and age-related photoreceptor density declines have been found in other species of birds [17], [69]. Photoreceptor degeneration can be accelerated due to low levels of carotenoids [70], which are obtained through the diet for many animals, including birds [71]. Nutrition during development could also affect photoreceptor morphology [72], and ambient light intensity has also been shown to affect retinal development [73]. Our intention was to estimate natural levels of variation in cone density in a wild population of birds; hence, it is likely that individuals varied in many of these factors.

We found between-individual differences in estimates of chromatic contrast of the wingbar, a signal that could affect competition and mate choice in house sparrows. Our findings reflect a scenario in which this signal is perceived by receivers in relation to the surrounding plumage as the visual background (within-signal contrast) rather than other objects as the background (e.g., vegetation). This reflects the way house sparrows displays to competitors or mates; for instance, they bow down and lift their wings so that their wingbars face the receivers [37]. Bókony *et al*. [50] showed that variation in the spectral properties of an individual's wingbar, versus the lesser and greater coverts, can influence his success at defending a resource. In our study, not only did the wingbars themselves differ in chromatic contrast, but the 26 subjects, when modeled as viewing these wingbars, differed in the degree to which the wingbars would appear to contrast chromatically with the other coverts. The range of variation in chromatic contrasts across individuals for a given wingbar could be considered small (16%). This finding suggests that intraspecific differences existed in the visual substrate, which is an important component of visual perception. However, it is not clear how much this different in visual contrast would influence behavior, which is should be addressed in the future. Depending on how different individuals perceive the chromatic contrast of these signals, they could make different decisions about whether, or how persistently, to attack a resource-holder. The wingbar has also been linked to female preferences [38], and may be an honest indicator of some aspects of condition [40], as parasitic chewing lice preferentially create holes in the white wingbar [39]. Thus, individual females with higher visual resolution would be able to better gauge the infection of a potential mate based on the detection of these small morphological irregularities. This could lead to an assortative mating scenario where females with finer visual resolution mate with high-quality males.

A visual signal may have multiple components that contain different types of information (chromatic and achromatic contrast, patterns, spatial resolution etc.), and consequently receivers may use different visual dimensions to assess the signal [74]. Our study addressed individual perceptual variation in two of these components: chromatic contrast and spatial resolution. We found that individuals that would have the potential to perform best in chromatic contrast would not necessarily have a similar or opposite performance in visual resolution (but see aforementioned caveats regarding the estimation of visual resolution). This could be because the density of photoreceptors influences the two modeled visual tasks through different processes: chromatic contrast through the *relative* representation of different photoreceptors (i.e., opponency channels, [34]) and visual resolution through the distance between photoreceptors (e.g., cell packing, [57]). Selection may favor some individuals assessing specific components of visual signals in certain contexts (e.g., agonistic interactions), and favor other individuals assessing other components of visual signals in other contexts (e.g., mating). This type of balancing selection could contribute to maintain the phenotypic variability in the visual sensory system we observed.

Between-individual variation in the visual sensory system has broad consequences in multiple scenarios beyond agonistic interactions and mating, such as anti-predator and foraging contexts. For instance, we speculate that differences in visual perception could help explain variation in risk-taking behavior: individuals that can detect predators sooner may be those that react more quickly to them, often interpreted as 'shy' in animal personality research [75]. Similarly, individual differences in provisioning rates [76] could stem from sensory differences in the abilities to detect food for offspring. In foraging contexts, individuals with higher visual resolution may have generally higher food detection rates, which may influence their tendency to be producers (i.e., search for food) rather than scroungers (i.e., exploit producers) during social foraging [77].

Our results have also important theoretical implications. Recent work has shown that when the ability to process signals with the peripheral sensory systems vary between receivers, model predictions can change [28], [78]. For example, individuals with greater sensory resolution than others may be able resolve finer differences between the signals of potential mates or rivals and thus assign rankings more distinctly before making decisions, ultimately affecting their fitness. Additionally, between-individual variation in resource-use could lead to niche specialization, a common phenomenon in many taxa [4], either due to spatial heterogeneity of food item types, or of the quality of ambient light that can alter the chromatic contrast of food items [79]. For instance, models considering species- and habitat-specific visual characteristics find that small changes in the sensory system can lead to large differences in prey perception and consequently food consumption [80], [81]. Overall, between-individual variation in sensory systems is important to consider as an underpinning of consistent individual differences in behavior or niche specialization.

## Conclusions

Individual variation is widespread across many taxa, and is important for evolution. However, variation in sensory systems has not received the attention it deserves, and often it is assumed that individuals within a population do not differ in the way that they detect stimuli. We found substantial between-individual variation in cone photoreceptor densities, a key element in the detection of visual stimuli. Further, through perceptual models, we found that this variation had the potential to generate variation in color vision and visual resolution. Functionally, chromatic contrast and visual resolution are two fundamental visual processes associated with mate choice, agonistic interactions, foraging, and predator avoidance. We argue that a broad array of evolutionary and ecological processes could be informed by assessing the degree of intraspecific variation in sensory systems.

## Supporting Information

Appendix S1 **Orientation of retinal landmarks, oil droplet identification, and stereological estimates.** This file describes the methods used to determine the location of the house sparrow fovea and the sampling area, as well as stereological estimates.
(PDF)

Appendix S2 **Chromatic contrast models, and spectral sensitivity parameters.** This file presents our chromatic contrast calculations with the Vorobyev and Osorio (1998) model, and describes the spectral sensitivity parameters that we used in our calculations.
(PDF)

Appendix S3 **Analyses of visual resolution considering axial length.** We explain three analyses of spatial resolving power conducted to support our use of the average axial length measures for missing data points, as well as the fixed effects from the model that was used for the results in the main text.
(PDF)

Appendix S4 **Distributions of residuals in models of proportions and ratios.** This file contains a description of the distribution of residuals from the models of cone type proportions and ratios, including images of the conditional residuals.
(PDF)

Appendix S5 **Average, minimum, and maximum cell counts and densities per retina, as well as cone type proportions.**
(PDF)

Appendix S6 **Fixed effects in mixed models of absolute cone densities.** Detailed results are presented on analyses of absolute cone densities, including the significance and estimates of the fixed effects (sex, eye, eccentricity, eccentricity × eye, date sampled, and observer) in the mixed models of absolute cone densities, as well as a discussion of the significant observer effect. This appendix also contains information on tests of the effects of eye measures (axial length, transverse length, and corneal diameter) on cell densities.
(PDF)
