# Supplementary material for: Individual Variation in Cone Photoreceptor Density in House Sparrows: Implications for Between-Individual Differences in Visual Resolution and Chromatic Contrast
Source: PLoS One. 2014 Nov 5;9(11):e111854. doi: 10.1371/journal.pone.0111854 (PMC4221115; doi:10.1371/journal.pone.0111854)
Supplement: Appendix S1 — Orientation of retinal landmarks, oil droplet identification, and stereological estimates. This file describes the methods used to determine the location of the house sparrow fovea and the sampling area, as well as stereological estimates. (PDF) [file pone.0111854.s001.pdf]

Appendix S1 - Orientation of retinal landmarks, oil droplet identification, and stereological estimates

In order to define the location of the sampling area in each of the retinas before counting cones, we first needed to determine the orientation of the house sparrow retina and the location the fovea (Figs. S1.1 and S1.2).

Orientation of the retina. The orientation of the retina can be measured in relation to the position of the bill and the pecten, which is a non-sensory structure that provides nutrients to the retina (Figs. S1.1 and S1.2). The retina is then oriented in terms of two axes: dorsal-ventral and nasal-temporal. To determine the angle of the dorsal-ventral axis from the pecten in house sparrows, we hemisected four left eyes and three right eyes while they were still in the skull. From photographs of the eyecup in the skull, we used Adobe Illustrator to measure the angle of the pecten to the dorsal-ventral axis. The mean angle was 34° ± 2.4°. We used this angle to orient the 52 retinas of the subjects in this study.

Location of the fovea in relation to the pecten and center of the retina. We located the average position of the house sparrow fovea using the distance and angle of the fovea from the tip of the optic nerve by combining two sources of data. First, we used images of six left eyes and five right eyes that we hemisected (Fig. S1.1). The house sparrow fovea is visible as a light pink spot under a dissecting scope when an eye is hemisected, before extraction of the retina from the eye cup (Fig. S1.1). Second, we used images from retinas that we whole-mounted and stained with cresyl violet (two left and two right eyes, Fig. S1.2), following methods in Ullmann *et al*. [1]. The fovea can be seen under a dissecting scope as an unstained area, since cresyl violet stains retinal ganglion cells which are absent at the fovea. All images were analyzed with ImageJ (http://rsbweb.nih.gov/ij/). We found that the fovea was on average 1,404 ± 15 μm from the pecten tip, and at a 98° ± 1° angle from the pecten. We used these averages to position the fovea on all retinas in this study. Details of the methods for these two sources of data are below.

Cartesian coordinates of the fovea. We used one of the whole-mounted and stained retinas to estimate the Cartesian coordinates of the fovea, using a recently published method [2]. To find the center of the retina, we fitted a circle with a diameter extending the widest width of the retina but minimizing the negative space between the retina and the circle (Fig. S1.2). We calculated r as the distance to the fovea from the center divided by the radius of the circle, which standardized this distance as a proportion. We determined the angle from the nasal portion of the retina to the fovea in radians (Θ), and calculated the x-coordinate as $r\text{cosine}(\Theta)$, and the y-coordinate as $r\text{sine}(\Theta)$. For this house sparrow retina (Fig. S1.2), the fovea was displaced from the center toward the temporal and dorsal sides of the retina with Cartesian coordinates of -.22, +.04.

Oil droplet identification. We used criteria given by Hart [3] to identify oil droplets, based on colour, size, and plane in the retina (Fig. S1.3). A total of four observers counted oil droplets after passing a training period on 83 sites, where successful training required a repeatability >0.90. In blocks of three to five birds (six to 10 retinas), sites were randomized and counted in this random order, with each observer counting a different set of randomized sites.

Stereological estimates. Our stereological procedures (see main text) resulted in the following stereological estimates [4-6] for the sampled perifoveal region across the 52 retinas (mean ± SEM): grid size (size of each image) = 0.30 ± 0.004 mm^2, total number of sites within the perifoveal region = 27 ± 0.47, total number sites counted within the perifoveal region = 20 ± 0.65, area sampling fraction (asf) = 0.008 ± 0.0001, total number of cells counted within the perifoveal region ($\sum Q^{-}$) = 3,378 ± 114.9, number of cells per site = 163 ± 3.2, observed coefficient of variation of group mean (CV) = 0.29 ± 0.01. We manually calculated two parameters of stereological reliability of our estimates. First, we calculated the Sheaffer-Mendenhall-Ott coefficient of error (CE), where values <0.1 are considered highly reliable [4]; our average (± SEM) CE across the 52 retinas = 0.068 ± 0.004. Second, we calculated the Sheaffer-Mendenhall-Ott CE^2/CV^2, which indicates the degree to which the variance in cell counts is due to sampling error caused by stereological procedures, where a value of <0.5 is considered highly reliable [4]; our average CE^2/CV^2 = 0.05 ± 0.002 (SEM), and all values were < 0.12, indicating that our estimates were acceptably reliable.

Supplementary Figures

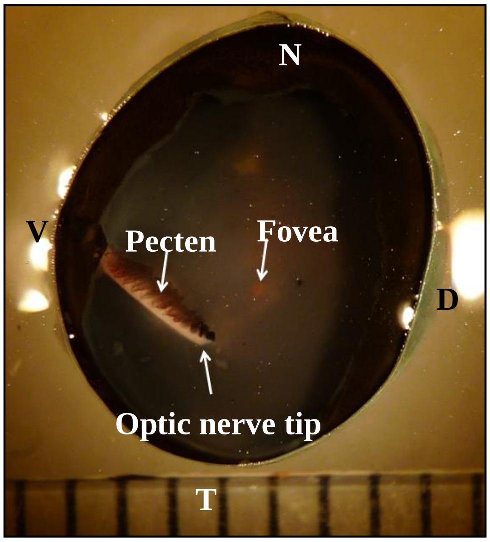

Figure S1.1. Orientation of the house sparrow retina in a hemisected left eye. D = dorsal; V = Ventral; N = Nasal; T = Temporal. The fovea is the light pink spot at the tip of the arrow, and the pecten is dark the filament structure attached to the white optic nerve. The ruler marks are mm.

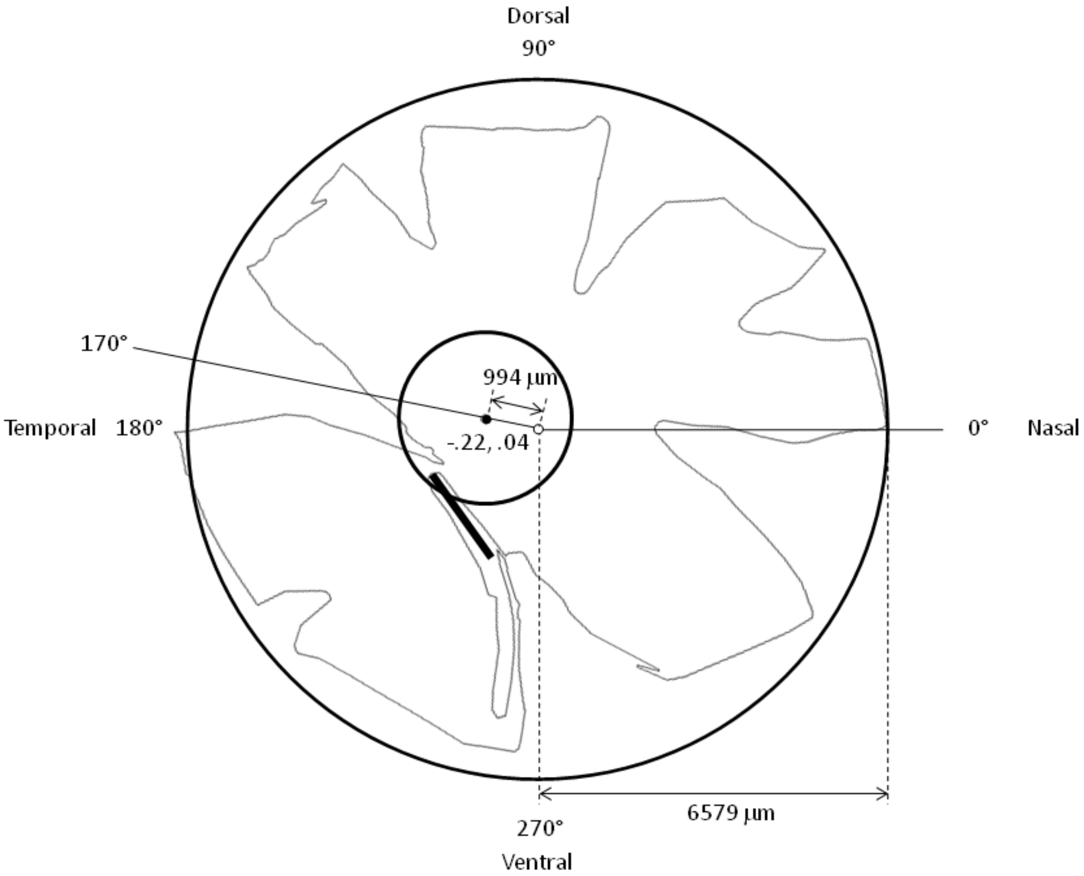

Figure S1.2. Schematic representation of a representative house sparrow right eye retina, showing the approximate sampling area. Determination of the Cartesian coordinates of the fovea followed [2]. This retina was stained with cresyl violet for retinal ganglion cells to establish the position of the fovea (indicated by filled black circle), which was located 994 µm from the center of the retina (indicated by open circle), at 170° from the nasal-temporal axis of the retina. The pecten is indicated by the thick black line. The approximate area sampled in our study (1600 µm radius around the fovea) is indicated by the bold circle centered on the fovea.

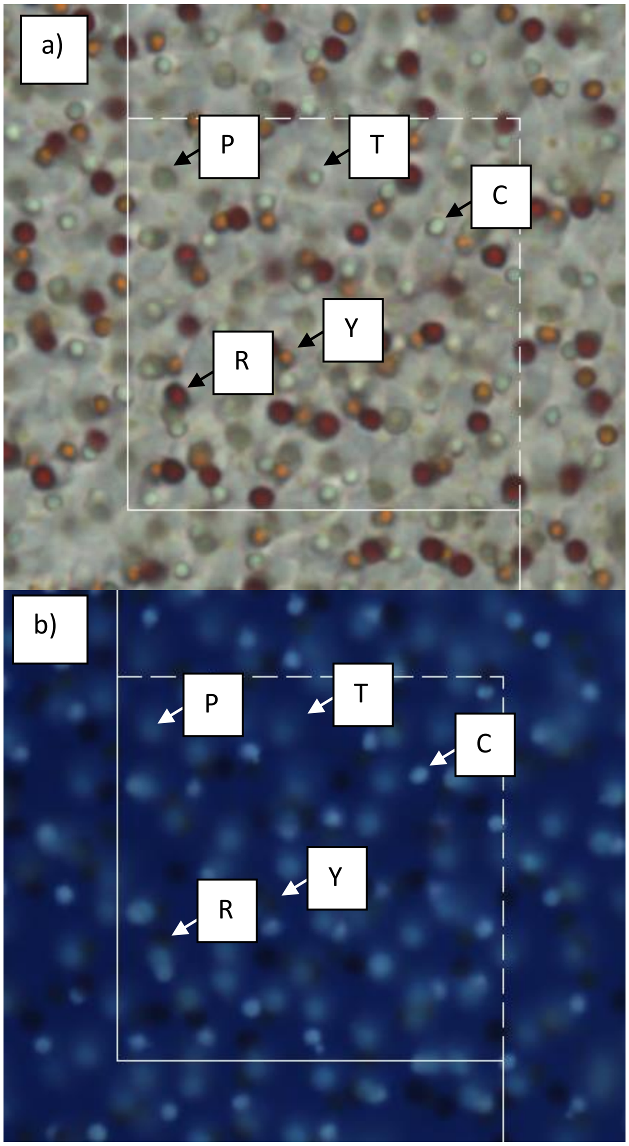

Figure S1.3. The appearance of oil droplets in a house sparrow retina. a) bright-field image, showing dull greenish P-types, bright light blue T-types and C-types, yellow Y-types, and red R-types. b) Epi-fluorescent image indicating the same oil droplets as in the bright-field image. The absence of the T-type allowed us to distinguish it from the C-type.

References

1. Ullmann JFP, Moore BA, Temple SE, Fernández-Juricic E, Collin SP (2012) The retinal wholemount technique: A window to understanding the brain and behaviour. Brain Behav Evol 79: 26-44.
2. Moore BA, Kamilar JM, Collin SP, Bininda-Emonds ORP, Dominy NJ, et al. (2012) A novel method for comparative analysis of retinal specialization traits from topographic maps. Journal of Vision 12: #13.
3. Hart NS (2001) Variations in cone photoreceptor abundance and the visual ecology of birds. J Comp Physiol A -Neuroethol Sens Neural Behav Physiol 187: 685-697.
4. Glaser EM, Wilson PD (1998) The coefficient of error of optical fractionator population size estimates: a computer simulation comparing three estimators. J Microsc 192: 163-171.
5. Slomianka L, West MJ (2005) Estimators of the precision of stereological estimates: An example based on the CA1 pyramidal cell layer of rats. Neuroscience 136: 757-767.
6. West MJ (2013) What to report: Information to be included in the publication of a stereological study. Cold Spring Harb Protoc 10.1101/pdb.top071894: 815-819.
