# Supplementary material for: Individual Variation in Cone Photoreceptor Density in House Sparrows: Implications for Between-Individual Differences in Visual Resolution and Chromatic Contrast
Source: PLoS One. 2014 Nov 5;9(11):e111854. doi: 10.1371/journal.pone.0111854 (PMC4221115; doi:10.1371/journal.pone.0111854)
Supplement: Appendix S2 — Chromatic contrast models, and spectral sensitivity parameters. This file presents our chromatic contrast calculations with the Vorobyev and Osorio (1998) model, and describes the spectral sensitivity parameters that we used in our calculations. (PDF) [file pone.0111854.s002.pdf]

Appendix S2. Chromatic contrast models, and spectral sensitivity parameters.

We used Avicol v6 [1] to calculate chromatic contrasts using the photon catch photoreceptor noise-limited model of Vorobyev and Osorio [2]. This model incorporated four components: 1) the reflectance spectra of the two object to be compared (wingbar and surrounding plumage, described in the main text), 2) the irradiance spectra of ambient light (described below), 3) the wavelength-specific spectral sensitivities of the house sparrow's single cone photoreceptors (described below), and 4) the relative cone densities (described in the main text). We modelled chromatic contrast for each retina separately (N=52), where all data inputs were the same for all retinas except the cone densities.

Chromatic contrast model

In the Vorobyev & Osorio [2] model, the square of chromatic contrast (ΔS) of the object against a background in a tetrachromatic colour space, like the one in birds, is defined as:

$$(\Delta S)^2 = ((e_1e_2)^2(\Delta f_4 - \Delta f_3)^2 + (e_1e_3)^2(\Delta f_4 - \Delta f_2)^2 + (e_1e_4)^2(\Delta f_3 - \Delta f_2)^2 + (e_2e_3)^2(\Delta f_4 - \Delta f_1)^2 + (e_2e_4)^2(\Delta f_3 - \Delta f_1)^2 + (e_3e_4)^2(\Delta f_2 - \Delta f_1)^2)/((e_1e_2e_3)^2 + (e_1e_2e_4)^2 + (e_1e_3e_4)^2 + (e_2e_3e_4)^2) \quad (1)$$

where e_i is the overall noise for photoreceptor class i, and Δf_i is the relationship between the quantum catch of two stimuli (a and b) for photoreceptor class i:

$$\Delta f_i = \ln(Q_{i,a}) - \ln(Q_{i,b}) = ln\left(\frac{Q_{i,a}}{Q_{i,b}}\right) \quad (2)$$

and

$$Q_{i,x} = \int_{\lambda_{min}}^{\lambda_{max}} R_x(\lambda)S_i(\lambda)I(\lambda)d(\lambda) \quad (3)$$

where λ is wavelength, $R_x(\lambda)$ is the wavelength-specific reflectance spectrum of the object x, $S_i(\lambda)$ is the wavelength-specific spectral sensitivity of receptor i, and $I(\lambda)$ is the wavelength-specific spectrum of ambient light. Integration is over the visible spectrum of the organism (from 300 to 700 nm for birds).

The noise (e_i) is defined as:

$$e_i = v_i/\sqrt{\eta_i} \quad (4)$$

where the standard deviation of the noise of a single photoreceptor cell is represented by v_i (the Weber fraction for the UVS cone, set at 0.05 in this study), and η_i is the density of photoreceptors of type i relative to the UVS densities.

We measured irradiance (spectral properties of ambient light) with an Ocean Optics JAZ spectrometer (Ocean Optics, Inc., FL) in West Lafayette, IN, USA, on an overcast day in June under a shaded area near a building, because it is a common location for mating and agonistic interactions. We took a total of nine irradiance measures in that location: one pointing directly vertically toward the sky (light source), four at a 45° angle from vertical (North, South, East, West), and four at a 90° angle from vertical (one at each cardinal point). The spectrometer measured irradiance from 190 nm to 891 nm, in approximately 0.382 nm increments, in units of µWatts/cm^2/nm, using a cosine corrector. We (1) averaged the nine measures at each wavelength, (2) interpolated the average spectrum 1 nm increments from 390 nm to 700 nm, and (3) converted the units to µmol/s/m^2 for use in chromatic contrast calculations.

Cone spectral sensitivities ($S_i(\lambda)$) are the result of a combination of the spectral absorbance of the visual pigment and the transmittance of the associated oil droplet [3]. The peak

sensitivity of visual pigments is characterized by λ_{max} [4], and we obtained these values from Table 2 in Hart and Hunt [5]. For house sparrows (*Passer domesticus*), Hart & Hunt [5] provide λ_{max} for SWS (445 nm), MWS (503 nm), and LWS (563 nm), but not UVS, so for the UVS cone we used the λ_{max} value for *Neochmia modesta* (373 nm), the most closely related species to house sparrows in that reference.

Oil droplet spectral sensitivities can be characterized by λ_{cut} (the wavelength at which 100% of the light is absorbed by the oil droplet) and β_{mid} (the slope of the tangent line at the wavelength where 50% of the light is absorbed). No data were available in the literature on house sparrow oil droplet parameters, so we used Microspectrophotometry (MSP) [6] to obtain absorbance spectra on oil droplets from three house sparrows (none of them were subjects in this study) from the central region of their retinas where the fovea is located. We used the mean values of λ_{cut} and β_{mid} collected from these three house sparrows (Table S2.1). Details of our MSP methods and analysis procedure can be found in Fernández-Juricic et al. [7].

Table S2.1. Oil droplet spectral sensitivity parameters used in modeling chromatic contrasts.

Oil droplet type	Mean λ_{cut}	Mean β_{mid}	Number of oil droplets
R-type (LWS cone)	572	0.025	50
Y-type (MWS cone)	520	0.029	53
C-type (SWS cone)	421	0.039	65
P-type (Double cone*)	424	0.036	59

* Oil droplets only found in the principal member of the double cone.

1. Gomez D (2006) AVICOL, a program to analyse spectrometric data. v6 ed.
2. Vorobyev M, Osorio D (1998) Receptor noise as a determinant of colour thresholds. Proc R Soc Lond B Biol Sci 265: 351-358.
3. Bowmaker JK, Heath LA, Wilkie SE, Hunt DM (1997) Visual pigments and oil droplets from six classes of photoreceptor in the retinas of birds. Vision Res 37: 2183-2194.
4. Govardovskii VI, Fyhrquist N, Reuter T, Kuzmin DG, Donner K (2000) In search of the visual pigment template. Vis Neurosci 17: 509-528.
5. Hart NS, Hunt DM (2007) Avian visual pigments: Characteristics, spectral tuning, and evolution. Am Nat 169: S7-S26.
6. Liebman PA (1972) Microspectrophotometry of photoreceptors. Handbook of Sensory Physiology 2: 481-528.
7. Fernández-Juricic E, Ojeda A, Deisher M, Burry B, Baumhardt P, et al. (2013) Do male and female cowbirds see their world differently? Implications for sex differences in the sensory system of an avian brood parasite. PloS One 8: e58985.
