# Supplementary material for: Individual Variation in Cone Photoreceptor Density in House Sparrows: Implications for Between-Individual Differences in Visual Resolution and Chromatic Contrast
Source: PLoS One. 2014 Nov 5;9(11):e111854. doi: 10.1371/journal.pone.0111854 (PMC4221115; doi:10.1371/journal.pone.0111854)
Supplement: Appendix S3 — Analyses of visual resolution considering axial length. We explain three analyses of spatial resolving power conducted to support our use of the average axial length measures for missing data points, as well as the fixed effects from the model that was used for the results in the main text. (PDF) [file pone.0111854.s003.pdf]

Appendix S3. Analyses of visual resolution considering axial length

Calculations of anatomical spatial resolving power (a proxy of visual resolution) require, among other parameters, eye axial length measurements. However, eye axial length measurements were not available for some eyes (n=18), we analyzed between-individual variation in anatomical spatial resolving power in three different ways:

(1) including only those retinas with eye axial length measures (Table S3.1),

(2) for retinas with eye axial length measurements the actual value was used, but for retinas without axial length measures we substituted the values with the average eye axial length from the measured retinas (6.75 mm) (Table S3.1; results in main text), and

(3) using the average axial length for all measured retinas (Table S3.1).

Variances and repeatabilities were very similar across the three data sets, and all showed significant between-individual variation (Table S3.1). This suggests that between-individual variation in visual resolution was not due to between-individual differences in eye axial length. In fact, when all variation due to axial length was removed (i.e., dataset #3 where we used the average axial length for all retinas), the between-individual variation in visual resolution was just as high as for dataset #1. We therefore used in the text anatomical spatial resolving power calculated according to #2 above. The effects of the fixed variables are shown in Table S3.2. Similar to the results presented in Additional File 2, we found significant effects of eccentricity and observer, but not of sex, eye, or the interaction between eccentricity and eye (Table S3.2).

Tables

Table S3.1. Sample size, variance, repeatability (*r*), and likelihood ratio tests of analysis of between-individual variation in spatial resolving power, compared between the analyses.

Analysis	Number of birds	Number of retinas	Number of sites	Between-individual variance ± S.E.M	*r*	Likelihood ratio test	
						χ^2_1	P-value
1	18	34	661	0.23 ± 0.10	0.15	53.5	<0.001
2	26	52	1060	0.22 ± 0.08	0.15	98	<0.001
3	26	52	1060	0.21 ± 0.14	0.14	102	<0.001

Analysis: 1) only included actual eye axial lengths; 2) included actual eye axial lengths where available but the average axial length of 6.75 mm where missing; 3) all retinas were given the average axial length.

Table S3.2. The significance of fixed effects on spatial resolving power, calculated as in Analysis #2 above.

Variable	Category	LS means ± SE	F	df	p
Sex	Female	10.93 ± 0.14	3.09	1, 23.27	0.09
	Male	10.57 ± 0.14			
Eye	Left	10.78 ± 0.11	0.45	1, 1032	0.50
	Right	10.72 ± 0.11			
Eccentricity			92.31	1, 1031	<0.0001
Eccentricity x Eye			1.01	1, 1031	0.31
Observer			8.82	1, 1018	<0.0001
