# Supplementary material for: Individual Variation in Cone Photoreceptor Density in House Sparrows: Implications for Between-Individual Differences in Visual Resolution and Chromatic Contrast
Source: PLoS One. 2014 Nov 5;9(11):e111854. doi: 10.1371/journal.pone.0111854 (PMC4221115; doi:10.1371/journal.pone.0111854)
Supplement: Appendix S4 — Distributions of residuals in models of proportions and ratios. This file contains a description of the distribution of residuals from the models of cone type proportions and ratios, including images of the conditional residuals. (PDF) [file pone.0111854.s004.pdf]

Appendix S4. Distributions of residuals in models of proportions

A) Cone type proportions

For each cone type separately, we used mixed models to analyze the proportion of that cone type out of all cones. The fixed effects in the models were: eye, sex, eccentricity, eccentricity*eye, counter, and the number of days in captivity. The random effects in the models were the random individual intercept for each eye, and the covariance between them. We also split the residual variance by eye. All analyses were conducted in SAS v9.3.

Since these data were proportions, we were concerned about normality. We assessed the degree of deviation from normality of these models by visually inspecting the conditional residuals (Figs. S4.1 – S4.5). Double cones were normal, which is not surprising given their high proportion values. MWS, SWS, and UVS residuals were very close to normal. LWS cone proportion residuals deviated the most from normality, being slightly skewed toward higher proportions (several low-proportion outliers). We looked back at images of these outlier sites, and could not find a justification for removal of those data – the sites were intact; the low proportions seemed to be real.

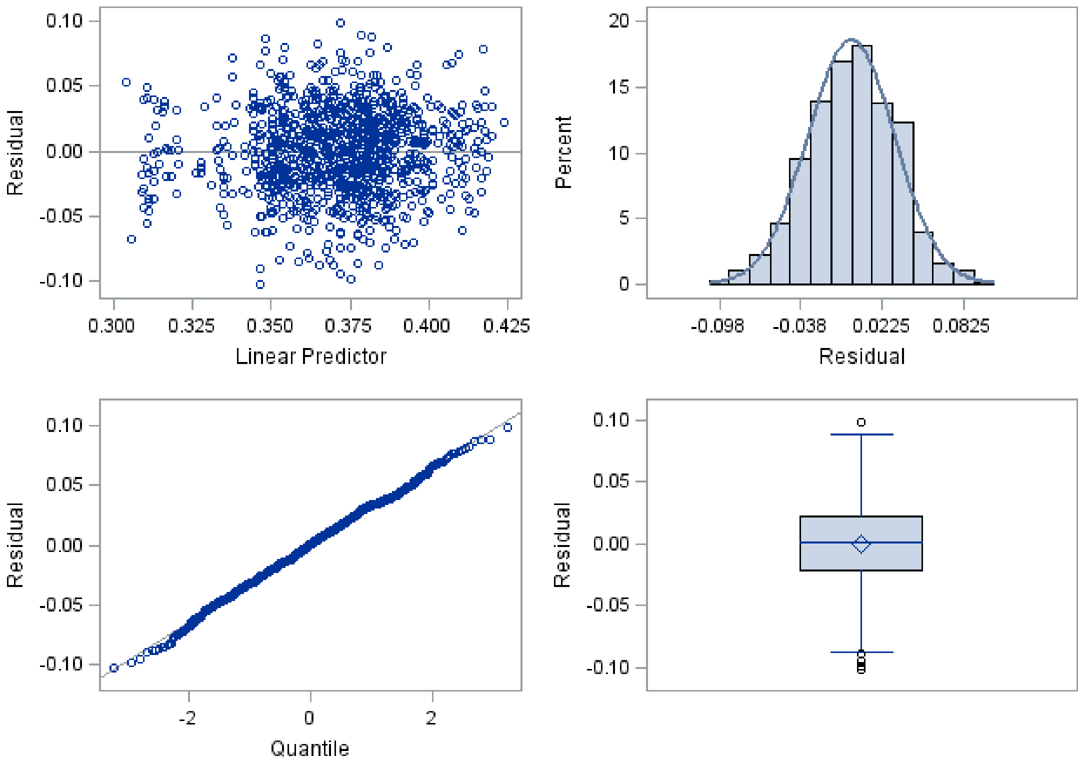

Figure S4.1 Conditional residuals from mixed models of the proportion of double cones.

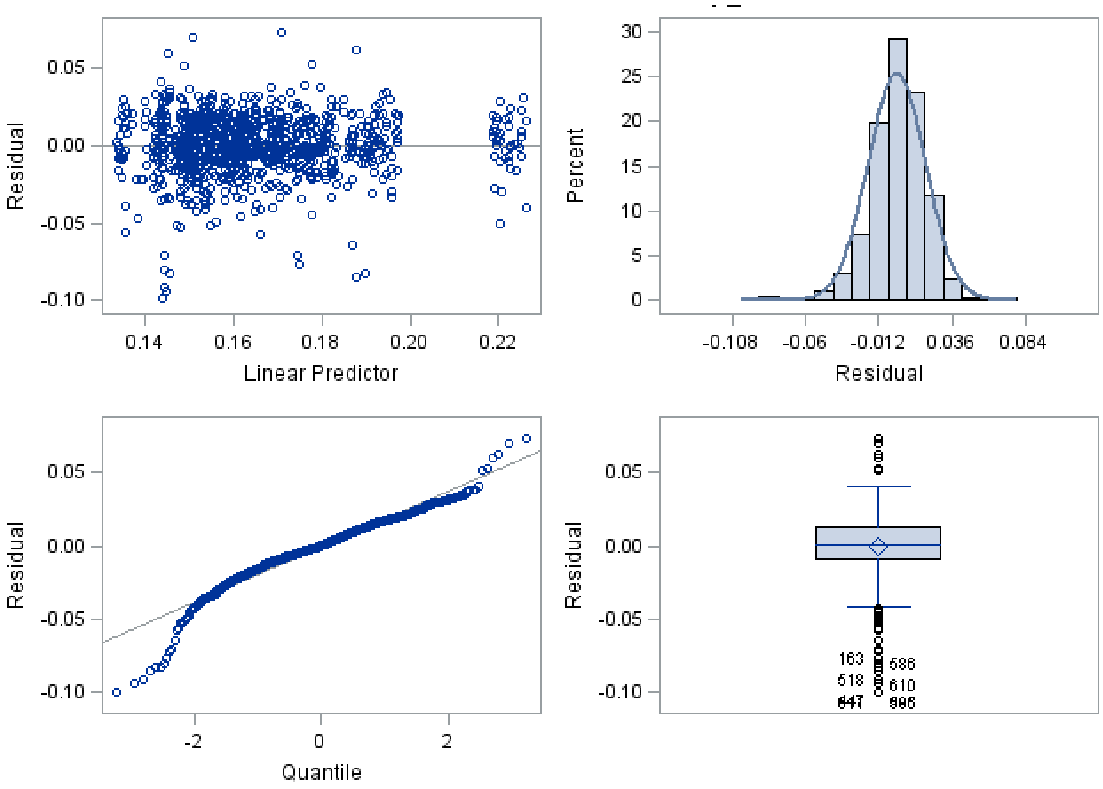

Figure S4.2 Conditional residuals from mixed models of the proportion of LWS cones.

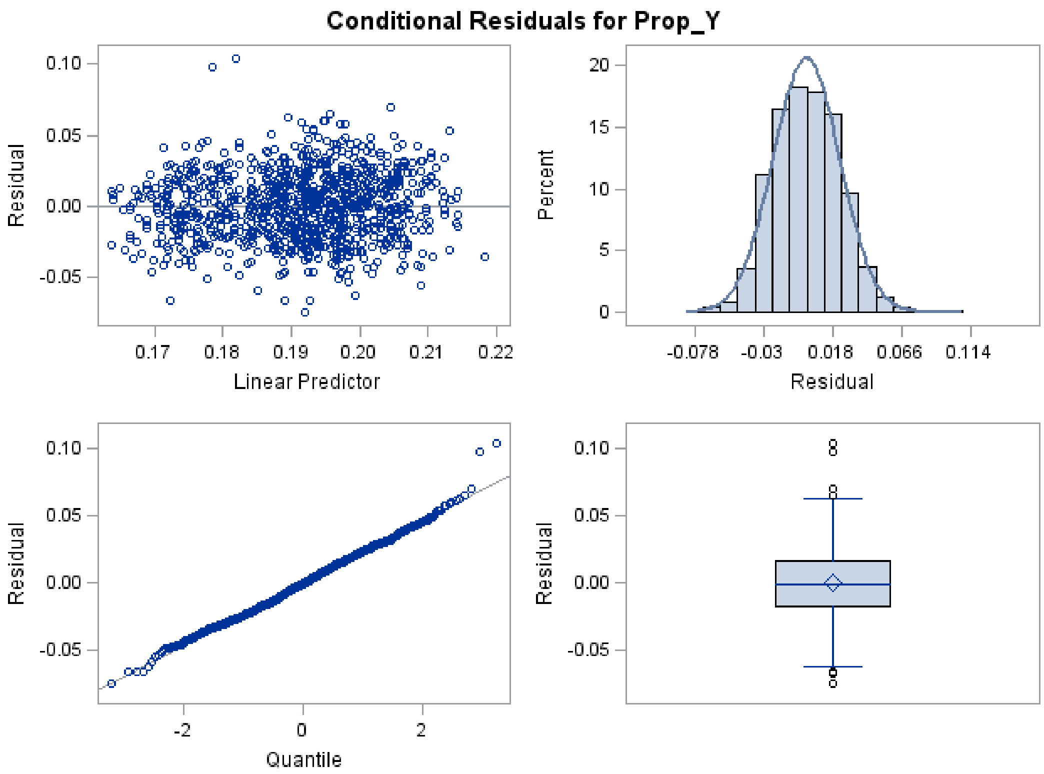

Figure S4.3 Conditional residuals from mixed models of the proportion of MWS cones.

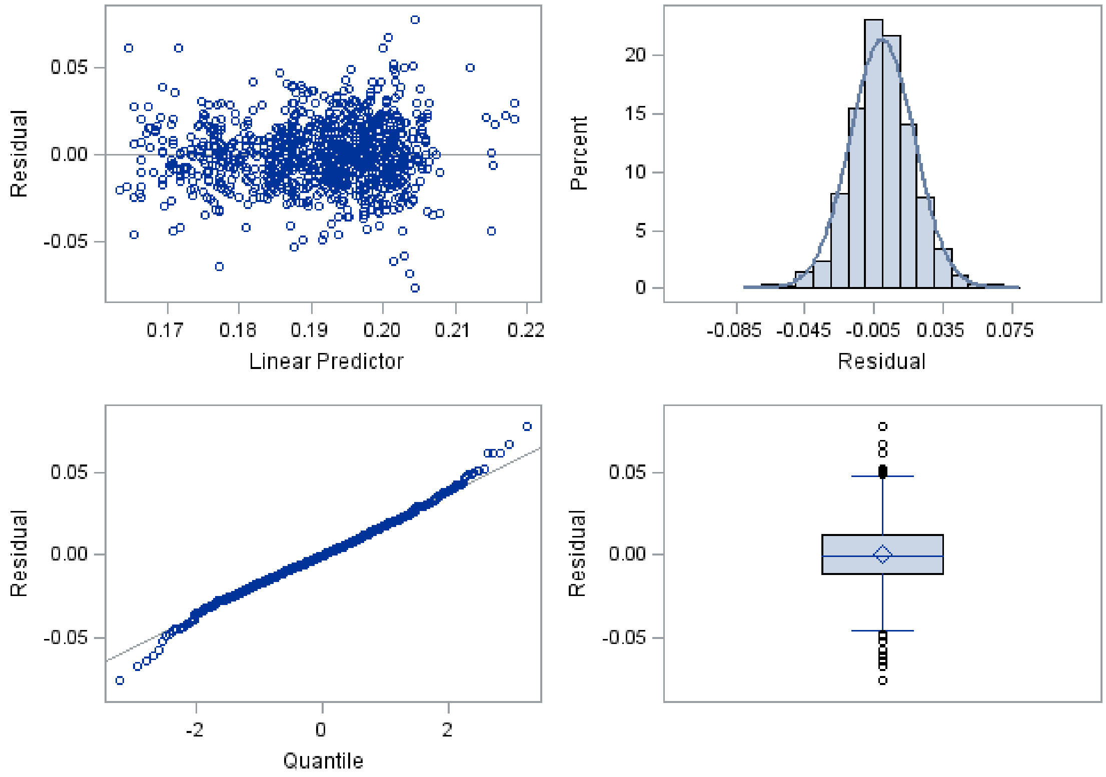

Figure S4.4 Conditional residuals from mixed models of the proportion of SWS cones.

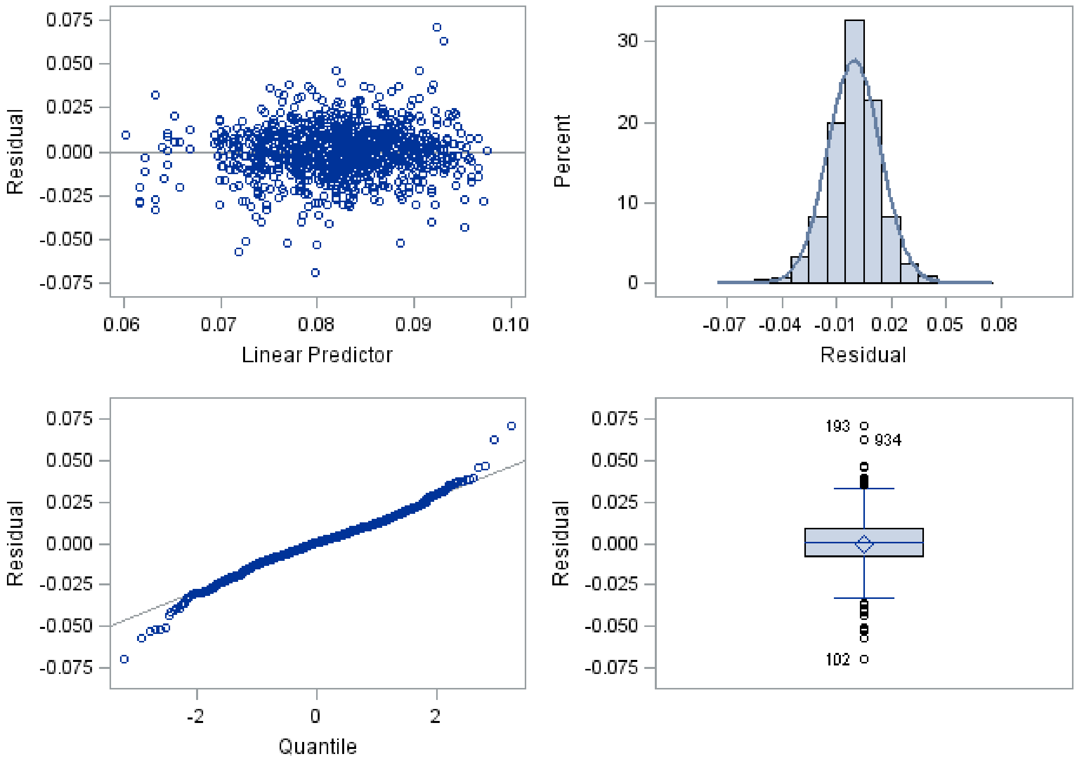

Figure S4.5 Conditional residuals from mixed models of the proportion of UVS cones.
