# Supplementary material for: Individual Variation in Cone Photoreceptor Density in House Sparrows: Implications for Between-Individual Differences in Visual Resolution and Chromatic Contrast
Source: PLoS One. 2014 Nov 5;9(11):e111854. doi: 10.1371/journal.pone.0111854 (PMC4221115; doi:10.1371/journal.pone.0111854)
Supplement: Appendix S5 — Average, minimum, and maximum cell counts and densities per retina, as well as cone type proportions. (PDF) [file pone.0111854.s005.pdf]

Appendix S5. Average, minimum, and maximum cell counts and densities per retina, as well as cone type proportions.

Table S5.1. Average, minimum, and maximum cell counts of the 52 retinas.

	Left				Right			
**Individual**	**Sites (N)**	**Minimum cell count**	**Average cell count**	**Maximum cell count**	**Sites (N)**	**Minimum cell count**	**Average cell count**	**Maximum cell count**
1	20	78	155	195	31	81	145	217
2	12	90	149	205	14	84	151	197
3	18	67	143	209	13	91	159	195
4	7	71	139	229	17	62	174	207
5	21	98	167	345	16	90	147	201
6	24	82	167	227	16	79	150	295
7	18	106	154	219	10	118	164	214
8	13	72	148	212	13	106	173	236
9	15	84	149	199	17	101	177	239
10	13	107	169	202	17	115	172	226
11	19	94	164	210	26	137	186	304
12	17	89	181	252	9	125	171	225
13	14	139	180	220	18	86	174	214
14	21	147	201	267	22	63	158	272
15	12	124	187	250	25	101	172	241
16	13	159	185	214	15	99	177	255
17	16	117	190	236	25	112	177	241
18	22	84	177	289	22	121	193	259
19	26	134	202	250	6	129	175	226
20	18	133	192	310	24	114	189	261
21	12	121	176	224	16	189	210	229
22	26	136	217	266	19	77	171	219
23	21	137	192	389	22	86	197	294
24	19	125	209	268	15	126	187	254
25	16	132	204	261	23	134	197	246
26	18	170	205	252	18	82	204	267
**All**	**451**	**67**	**177**	**389**	**469**	**62**	**175**	**304**

Data includes only the sites where the full 0.0025 mm^2 counting frame was used (N=920).

Table S5.2. Average, minimum, and maximum densities (cells/mm^2) of the 52 retinas.

	Left				Right			
Individual	Sites (N)	Minimum density	Average density	Maximum density	Sites (N)	Minimum density	Average density	Maximum density
1	22	31200	61164	78000	32	32400	59913	117600
2	19	36000	61312	83200	17	33600	62871	89600
3	21	26800	57594	83600	16	36400	63975	78000
4	9	28400	53704	91600	20	24800	69920	82800
5	23	39200	66533	138000	17	36000	59576	80400
6	26	32800	67231	90800	16	31600	59975	118000
7	18	42400	61756	87600	14	47200	64581	85600
8	18	28800	60030	84800	16	42400	69525	94400
9	15	33600	59733	79600	20	40400	69580	95600
10	17	42800	71859	92800	22	46000	71709	94400
11	21	37600	66013	93867	28	54800	74171	121600
12	19	35600	72105	100800	12	35200	67067	91200
13	20	39200	73680	107200	23	34400	71849	105600
14	25	58800	79408	106800	25	25200	61632	108800
15	16	36800	74225	100000	25	40400	68688	96400
16	14	63600	74086	85600	18	39600	71044	102000
17	23	46800	78632	112800	27	44800	70711	96400
18	24	33600	70550	115600	23	48400	77200	103600
19	28	53600	81395	100000	11	51600	71236	96000
20	23	44800	77635	124000	25	45600	76208	104400
21	18	44000	70370	89600	20	75600	84553	98400
22	28	54400	86281	106400	22	30800	71194	117333
23	24	54800	75628	155600	22	34400	78982	117600
24	19	50000	83474	107200	17	50400	73176	101600
25	16	52800	81525	104400	24	53600	79483	98400
26	19	68000	81747	100800	23	32800	82261	106800
**All**	**525**	**26800**	**71064**	**155600**	**535**	**24800**	**70426**	**121600**

Data includes all the sites (N=1060), with densities calculated based on the area sampled.

Table S5.3. Average, minimum, and maximum proportions of Double cones of the 52 retinas.

	Left				Right			
**Individual**	**Sites (N)**	**Minimum % Double**	**Average % Double**	**Maximum % Double**	**Sites (N)**	**Minimum % Double**	**Average % Double**	**Maximum % Double**
1	22	30%	37%	43%	32	28%	37%	44%
2	19	28%	36%	43%	17	27%	36%	41%
3	21	37%	41%	44%	16	35%	40%	45%
4	9	28%	35%	45%	20	34%	38%	44%
5	23	33%	38%	44%	17	33%	39%	43%
6	26	29%	38%	47%	16	33%	38%	44%
7	18	29%	35%	41%	14	28%	34%	44%
8	18	28%	38%	45%	16	30%	35%	40%
9	15	28%	39%	43%	20	27%	35%	41%
10	17	34%	39%	44%	22	36%	39%	42%
11	21	32%	38%	47%	28	34%	40%	45%
12	19	28%	37%	43%	12	32%	38%	43%
13	20	31%	38%	43%	23	24%	36%	44%
14	25	33%	36%	40%	25	28%	35%	41%
15	16	32%	36%	40%	25	34%	38%	41%
16	14	30%	33%	40%	18	29%	34%	42%
17	23	30%	38%	47%	27	30%	38%	45%
18	24	33%	39%	45%	23	31%	37%	47%
19	28	30%	38%	44%	11	29%	38%	45%
20	23	32%	37%	42%	25	32%	37%	43%
21	18	26%	35%	41%	20	30%	36%	42%
22	28	35%	42%	50%	22	32%	39%	47%
23	24	26%	38%	46%	22	36%	40%	45%
24	19	30%	35%	41%	17	31%	35%	38%
25	16	30%	34%	39%	24	30%	35%	41%
26	19	27%	31%	35%	23	24%	31%	37%
**Overall**	**525**	**26%**	**37%**	**50%**	**535**	**24%**	**37%**	**47%**

Data includes all the sites (N=1060)

Table S5.4. Average, minimum, and maximum proportions of LWS cones of the 52 retinas.

	Left				Right			
**Individual**	**Sites (N)**	**Minimum % LWS**	**Average % LWS**	**Maximum % LWS**	**Sites (N)**	**Minimum % LWS**	**Average % LWS**	**Maximum % LWS**
1	22	11%	15%	22%	32	10%	15%	19%
2	19	10%	17%	21%	17	16%	18%	21%
3	21	13%	16%	18%	16	13%	17%	20%
4	9	11%	15%	18%	20	13%	16%	19%
5	23	12%	16%	19%	17	14%	17%	23%
6	26	11%	15%	20%	16	11%	14%	19%
7	18	15%	18%	23%	14	15%	18%	21%
8	18	10%	18%	25%	16	16%	20%	24%
9	15	5%	14%	17%	20	13%	16%	19%
10	17	14%	16%	18%	22	14%	16%	18%
11	21	4%	15%	20%	28	5%	14%	18%
12	19	12%	16%	18%	12	14%	16%	18%
13	20	10%	16%	18%	23	11%	16%	24%
14	25	15%	17%	19%	25	13%	17%	20%
15	16	16%	18%	20%	25	16%	18%	21%
16	14	18%	20%	23%	18	11%	19%	22%
17	23	11%	15%	18%	27	13%	15%	18%
18	24	11%	14%	17%	23	11%	15%	18%
19	28	11%	16%	19%	11	13%	16%	20%
20	23	10%	18%	20%	25	16%	18%	22%
21	18	13%	16%	19%	20	15%	17%	20%
22	28	8%	14%	17%	22	9%	14%	16%
23	24	13%	16%	26%	22	12%	16%	17%
24	19	14%	17%	19%	17	14%	16%	20%
25	16	16%	19%	21%	24	17%	19%	22%
26	19	19%	23%	26%	23	17%	22%	25%
**Overall**	**525**	**4%**	**16%**	**26%**	**535**	**5%**	**17%**	**25%**

Data includes all the sites (N=1060)

Table S5.5. Average, minimum, and maximum proportions of MWS cones of the 52 retinas.

Individual	Left				Right			
	Sites (N)	Minimum % MWS	Average % MWS	Maximum % MWS	Sites (N)	Minimum % MWS	Average % MWS	Maximum % MWS
1	22	15%	19%	25%	32	15%	21%	25%
2	19	15%	20%	24%	17	16%	21%	27%
3	21	13%	17%	23%	16	13%	18%	21%
4	9	16%	20%	26%	20	13%	18%	22%
5	23	14%	17%	22%	17	13%	17%	20%
6	26	12%	19%	25%	16	16%	20%	25%
7	18	16%	20%	23%	14	15%	20%	25%
8	18	14%	19%	24%	16	16%	20%	22%
9	15	16%	19%	22%	20	17%	20%	24%
10	17	17%	20%	23%	22	16%	19%	23%
11	21	18%	22%	27%	28	14%	19%	25%
12	19	15%	19%	25%	12	15%	18%	21%
13	20	12%	18%	23%	23	14%	19%	23%
14	25	16%	19%	23%	25	14%	19%	24%
15	16	16%	19%	21%	25	14%	18%	21%
16	14	17%	20%	23%	18	17%	20%	24%
17	23	15%	19%	26%	27	14%	19%	23%
18	24	16%	19%	24%	23	16%	19%	23%
19	28	17%	20%	25%	11	16%	20%	23%
20	23	13%	18%	23%	25	14%	17%	20%
21	18	16%	19%	24%	20	14%	20%	25%
22	28	13%	17%	28%	22	13%	18%	29%
23	24	11%	18%	22%	22	13%	17%	21%
24	19	17%	21%	24%	17	18%	21%	23%
25	16	15%	21%	25%	24	17%	20%	25%
26	19	15%	20%	24%	23	15%	20%	26%
**Overall**	**525**	**11%**	**19%**	**28%**	**535**	**13%**	**19%**	**29%**

Data includes all the sites (N=1060)

Table S5.6. Average, minimum, and maximum proportions of SWS cones of the 52 retinas.

	Left				Right			
Individual	Sites (N)	Minimum % SWS	Average % SWS	Maximum % SWS	Sites (N)	Minimum % SWS	Average % SWS	Maximum % SWS
1	22	13%	20%	28%	32	17%	20%	25%
2	19	11%	18%	23%	17	12%	17%	23%
3	21	15%	18%	22%	16	16%	18%	21%
4	9	17%	23%	26%	20	15%	20%	25%
5	23	17%	20%	22%	17	16%	19%	23%
6	26	17%	20%	25%	16	16%	20%	23%
7	18	13%	19%	22%	14	15%	21%	26%
8	18	12%	17%	19%	16	13%	17%	19%
9	15	18%	20%	26%	20	17%	20%	23%
10	17	15%	17%	21%	22	17%	19%	23%
11	21	17%	20%	24%	28	12%	19%	23%
12	19	15%	19%	23%	12	15%	18%	23%
13	20	16%	19%	21%	23	17%	20%	23%
14	25	17%	20%	22%	25	17%	20%	24%
15	16	16%	19%	23%	25	16%	19%	23%
16	14	16%	18%	21%	18	16%	19%	23%
17	23	16%	19%	23%	27	16%	20%	23%
18	24	15%	19%	23%	23	14%	20%	25%
19	28	15%	17%	21%	11	12%	17%	20%
20	23	16%	18%	23%	25	17%	20%	23%
21	18	17%	21%	24%	20	16%	18%	22%
22	28	16%	18%	23%	22	14%	19%	23%
23	24	17%	20%	24%	22	14%	19%	21%
24	19	16%	20%	23%	17	19%	20%	22%
25	16	14%	18%	20%	24	12%	16%	21%
26	19	16%	19%	23%	23	13%	18%	23%
**Overall**	**525**	**11%**	**19%**	**28%**	**535**	**12%**	**19%**	**26%**

Data includes all the sites (N=1060)

Table S5.7. Average, minimum, and maximum proportions of UWS cones of the 52 retinas.

	Left				Right			
Individual	Sites (N)	Minimum % UVS	Average % UVS	Maximum % UVS	Sites (N)	Minimum % UVS	Average % UVS	Maximum % UVS
1	22	5%	9%	13%	32	3%	7%	12%
2	19	6%	9%	13%	17	4%	9%	13%
3	21	4%	8%	11%	16	4%	7%	10%
4	9	2%	7%	10%	20	4%	8%	10%
5	23	7%	9%	13%	17	4%	8%	10%
6	26	1%	8%	11%	16	7%	9%	13%
7	18	6%	7%	10%	14	3%	7%	10%
8	18	6%	8%	11%	16	6%	8%	11%
9	15	7%	8%	10%	20	6%	9%	12%
10	17	5%	8%	11%	22	6%	8%	10%
11	21	3%	6%	10%	28	4%	7%	10%
12	19	7%	9%	10%	12	6%	10%	18%
13	20	5%	10%	16%	23	6%	9%	14%
14	25	6%	8%	11%	25	7%	9%	11%
15	16	7%	8%	10%	25	1%	7%	9%
16	14	7%	9%	10%	18	6%	8%	10%
17	23	6%	8%	10%	27	5%	8%	11%
18	24	6%	8%	10%	23	5%	8%	11%
19	28	5%	9%	12%	11	6%	9%	10%
20	23	5%	8%	11%	25	6%	9%	11%
21	18	4%	9%	11%	20	6%	9%	12%
22	28	7%	9%	11%	22	7%	10%	16%
23	24	6%	9%	11%	22	4%	8%	12%
24	19	4%	7%	9%	17	5%	7%	9%
25	16	3%	8%	12%	24	6%	9%	11%
26	19	5%	8%	12%	23	6%	8%	10%
**Overall**	**525**	**1%**	**8%**	**16%**	**535**	**1%**	**8%**	**18%**

Data includes all the sites (N=1060)
