# Supplementary material for: Individual Variation in Cone Photoreceptor Density in House Sparrows: Implications for Between-Individual Differences in Visual Resolution and Chromatic Contrast
Source: PLoS One. 2014 Nov 5;9(11):e111854. doi: 10.1371/journal.pone.0111854 (PMC4221115; doi:10.1371/journal.pone.0111854)
Supplement: Appendix S6 — Fixed effects in mixed models of absolute cone densities. Detailed results are presented on analyses of absolute cone densities, including the significance and estimates of the fixed effects (sex, eye, eccentricity, eccentricity × eye, date sampled, and observer) in the mixed models of absolute cone densities, as well as a discussion of the significant observer effect. This appendix also contains information on tests of the effects of eye measures (axial length, transverse length, and corneal diameter) on cell densities. (PDF) [file pone.0111854.s006.pdf]

Appendix S6. Fixed effects in mixed models of absolute cone densities

The results below are from general linear mixed models with absolute cone densities as the dependent variable. We ran seven models, where the dependent variables were the absolute densities of: 1) all cones, 2) double cones, 3) single cones combined, 4) LWS cones, 5) MWS cones, 6) SWS cones, and 7) UVS cones. Each model had the following fixed effects: sex, eye, eccentricity, eccentricity × eye, observer (who counted the oil droplets), and date the retina was extracted. LS Means and standard errors are given for sex and eye effects (Tables S6.1 through S6.7). Also, for a subset of the data for which we had eye measures, we tested whether axial length, transverse length, and corneal diameter explained cone densities (Table S6.8)

Results. Fixed effects for each type of cone can be found below in Tables S6.1 through S6.7. We did not find any evidence for effects of sex, eye, eccentricity × eye, or date sampled ($p>0.05$). Eccentricity was a significant factor explaining absolute cone densities for all cone types ($p<0.001$). Observer was also significant for all cone types ($p<0.05$). None of the measures of eye size were related to cone densities ($p>0.05$).

Discussion

It is expected that eccentricity would significantly affect all cone types, because in animals with a fovea cone densities are highest near the fovea (Walls, 1942).

The significant observer effects are unlikely to bias our results on between-individual variation in house sparrows. We controlled for inter-observer differences in two ways. First, A.L.E. extensively trained three additional observers to identify oil droplets. Training consisted of 83 training sites, and at the end of this process all three additional observers had repeatabilities >0.9 compared with A.L.E. Second, the order of sites, retinas, and birds were randomized before being assigned randomly to each observer. The randomization of counting orders and observer identity means that sites and observers were not organized in a way that was associated with any other fixed effect, and no observer counted an entire retina or eye. Because of this random dispersal of measurement error, it is unlikely that differences due to observer would have strong effects on variation between individual retinas or eyes.

Despite controlling for observer effects, we explored whether the variance due to observer contributed to the between-individual variance. Theoretically, random variance can be partitioned into between-individual variance and within-individual variance (the residual from the model after accounting for the variance due to fixed effects) (Fig. S6.1a, Model 1). When a fixed effect is added to the model (Fig. S6.1a, Models 2 and 3), it can account for some of the within-individual variance (Fig. S6.1a Model 2), and/or some of the within-individual variance (Fig. S6.1a Model 3).

Because we randomly assigned sites to observers (observers were not directly associated with an entire retina or bird), observer effects likely contributed more to the within-individual variation than the between-individual variation. To test this, we removed observer from the model of total absolute densities, and compared the between-individual variance in that model to the full model presented in the main text. If differences among observers biased our results toward higher between-individual variation than actually existed, we would expect the variance due to observer to be pulled from the between-individual variance, and for the between-individual variance to decrease when compared with the model without observer (Fig. S6.1a, compare Models 1 and 3). If observer did *not* bias our results in favour of more between-individual variation, then observer variance should pull from the within-individual variance, and within-individual variance should decrease in comparison to the model without observer.

We found that adding observer decreased the within-individual variance from 73.09% to 70.85% (a difference of 2.24%), but only decreased the between-individual variance by a negligible amount (0.25%) (Fig. S6.1b). This suggests that the differences between observers in oil droplet densities did not contribute much (< 1%) to the between-individual variation; hence, the significant between-individual variation that we found was not biased by observer differences. Furthermore, we accounted for the fixed effect of observer in our models, which would have removed any between-individual variation that was due to observer, and the repeatabilities were calculated based on the between-individual variation that was *not* due to observer.

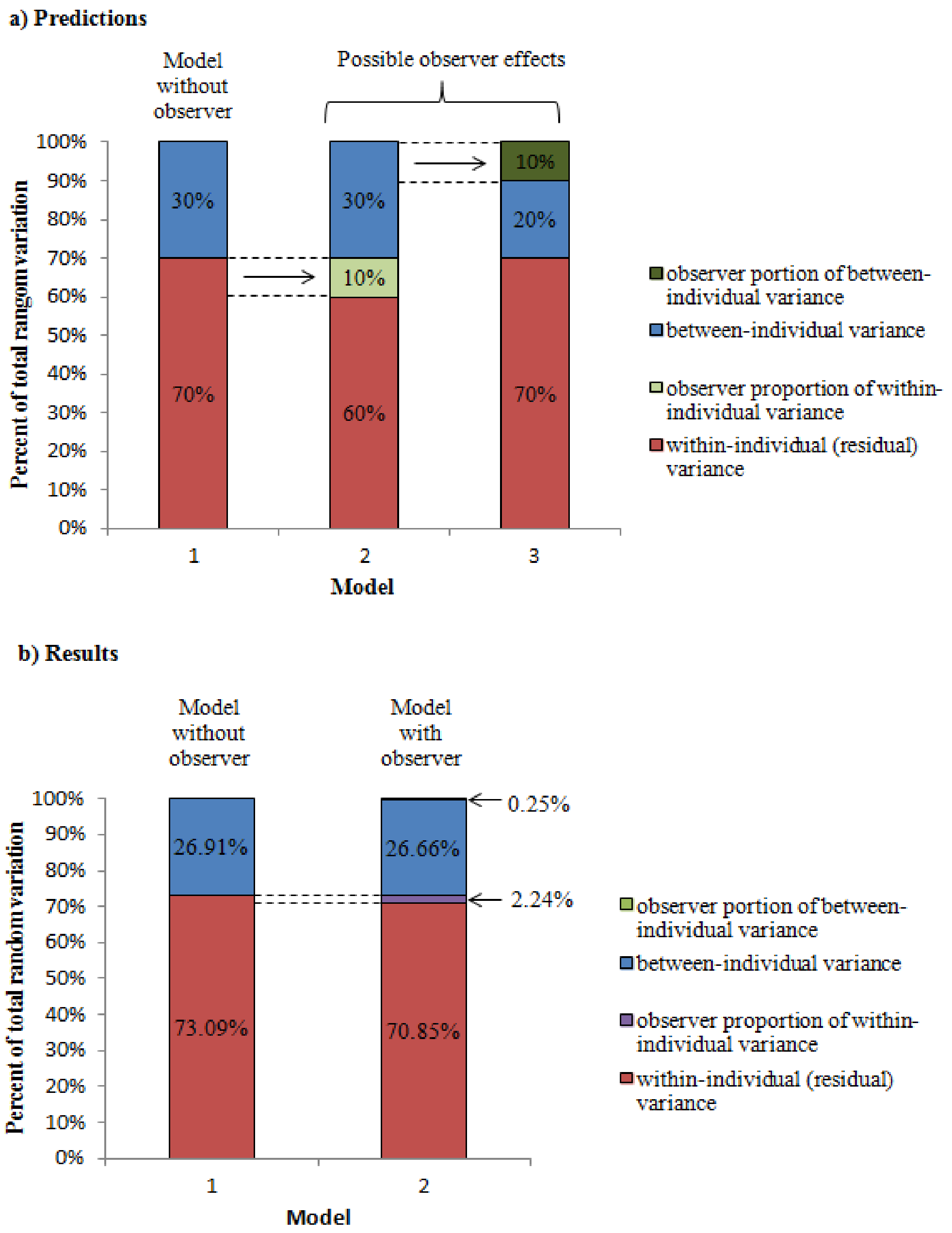

Figure S6.1. Comparisons of models with and without the fixed effect of observer, to determine if observer differences contributed more to between- or within-individual variance. (a) Theoretical graph to illustrate possible observer effects: Model 1) A hypothetical model without observer, where between-individual factors account for 30% of the total variance (blue), while within-individual factors account for 70% of the variance (red). Models 2 and 3 show the possible results when observer is added to the model. Model 2 shows a scenario where observer accounts for within-individual variance, and Model 3 shows a scenario where the observer

accounts for between-individual variance. (b) Our results, comparing the model without observer (Model 1) to the model with observer (Model 2), showed that observer accounted for more within-individual variation (2.24%) than between-individual variation (0.25%), supporting the contention that our between-individual significant effects are not likely biased by the observer effects.

References

Walls G.L. 1942 *The vertebrate eye and its adaptive radiation.* Bloomfield Hills, MI, Cranbrook Institute of Science.

Supplementary Tables

Table S6.1. The significance of fixed effects on the absolute total cone density.

Variable	Category	*Estimate	F	df	p
Sex	Female	71.79 ± 1.68	1.86	1, 23.14	0.19
	Male	68.33 ± 1.80			
Eye	Left	70.39 ± 1.69	0.01	1, 266.3	0.93
	Right	69.73 ± 1.35			
†Eccentricity		-0.0128 ± 0.0017	105	1, 1008	<0.001
Eccentricity x Eye		0.0008 ± 0.0024	0.12	1, 1013	0.73
Extraction date		0.0044 ± 0.0126	0.12	1, 26.98	0.73
Observer			11.8	3, 1038	<0.001

The dependent variable units were cells/1000mm^2;

†Eccentricity units were µm.

*Estimates: Sex and eye, LS means ± SE. Eccentricity and extraction date, partial β.

Eccentricity x Eye, the differential effect of the left eye compared to the right eye.

Table S6.2. The significance of fixed effects on the absolute density of double cones.

Variable	Category	*Estimate	F	df	p
Sex	Female	26.24 ± 0.99	0.28	1, 17.96	0.61
	Male	25.50 ± 0.99			
Eye	Left	25.89 ± 0.93	0.03	1, 167.0	0.87
	Right	25.86 ± 0.71			
†Eccentricity		-0.0057 ± 0.0007	125	1, 1006	<0.001
Eccentricity x Eye		0.0005 ± 0.001	0.26	1, 1011	0.61
Date sampled		0.0026 ± 0.0053	0.25	1, 26.68	0.62
Observer			12.4	3, 1037	<0.001

The dependent variable units were cells/1000mm^2;

†Eccentricity units were µm.

*Estimates: Sex and eye, LS means ± SE. Eccentricity and extraction date, partial β.

Eccentricity x Eye, the differential effect of the left eye compared to the right eye.

Table S6.3. The significance of fixed effects on the absolute density of single cones.

Variable	Category	*Estimate	F	df	p
Sex	Female	45.64 ± 1.29	2.49	1, 23.13	0.13
	Male	42.75 ± 1.29			
Eye	Left	44.35 ± 1.14	0	1, 291	0.97
	Right	44.04 ± 0.96			
†Eccentricity		-0.0071 ± 0.0011	74.9	1, 1010	<0.001
Eccentricity x Eye		0.0003 ± 0.0016	0.05	1, 1015	0.83
Date sampled		0.0018 ± 0.0091	0.04	1, 26.34	0.84
Observer			9.56	3, 1037	<0.001

The dependent variable units were cells/1000mm^2;

†Eccentricity units were μm.

*Estimates: Sex and eye, LS means ± SE. Eccentricity and extraction date, partial β.

Eccentricity x Eye, the differential effect of the left eye compared to the right eye.

Table S6.4. The significance of fixed effects on the absolute density of LWS cones.

Variable	Category	*Estimate	F	df	p
Sex	Female	5.97 ± 0.26	0.58	1, 18.08	0.46
	Male	5.70 ± 0.26			
Eye	Left	5.82 ± 0.24	0.01	1, 229.3	0.927
	Right	5.85 ± 0.18			
†Eccentricity		-0.0019 ± 0.0003	70	1, 1004	<0.001
Eccentricity x Eye		-0.00004 ± 0.00046	0.01	1, 1008	0.93
Date sampled		0.0015 ± 0.0039	0.14	1, 24.36	0.71
Observer			3.6	3, 1027	0.01

The dependent variable units were cells/1000mm^2;

†Eccentricity units were μm.

*Estimates: Sex and eye, LS means ± SE. Eccentricity and extraction date, partial β.

Eccentricity x Eye, the differential effect of the left eye compared to the right eye.

Table S6.5. The significance of fixed effects on the absolute density of MWS cones.

Variable	Category	*Estimate	F	df	p
Sex	Female	13.68 ± 0.44	0.33	1, 17.80	0.57
	Male	13.31 ± 0.44			
Eye	Left	13.48 ± 0.37	0.05	1, 264.2	0.827
	Right	13.50 ± 0.34			
†Eccentricity		-0.0017 ± 0.0004	32.5	1, 1013	<0.001
Eccentricity x Eye		0.0003 ± 0.0005	0.3	1, 1018	0.58
Date sampled		-0.0009 ± 0.0028	0.1	1, 26.69	0.76
Observer			5.67	3, 1040	<0.001

The dependent variable units were cells/1000mm^2;

†Eccentricity units were μm.

*Estimates: Sex and eye, LS means ± SE. Eccentricity and extraction date, partial β.

Eccentricity x Eye, the differential effect of the left eye compared to the right eye.

Table S6.6. The significance of fixed effects on the absolute density of SWS cones.

Variable	Category	*Estimate	F	df	p
Sex	Female	13.76 ± 0.39	0.51	1, 17.82	0.49
	Male	13.35 ± 0.39			
Eye	Left	13.54 ± 0.35	0.02	1, 219.9	0.877
	Right	13.57 ± 0.32			
Eccentricity		-0.002 ± 0.0004	69.2	1, 1016	<0.001
Eccentricity x Eye		0.0002 ± 0.0005	0.08	1, 1021	0.77
Date sampled		0.0002 ± 0.003	0.01	1, 26.64	0.93
Observer			13.1	3, 1040	<0.001

The dependent variable units were cells/1000mm^2;

†Eccentricity units were μm.

*Estimates: Sex and eye, LS means ± SE. Eccentricity and extraction date, partial β.

Eccentricity x Eye, the differential effect of the left eye compared to the right eye.

Table S6.7. The significance of fixed effects on the absolute density of UVS cones.

Variable	Category	*Estimate	F	df	p
Sex	Female	12.09 ± 0.71	0.44	1, 18.04	0.51
	Male	11.41 ± 0.71			
Eye	Left	11.60 ± 0.54	0.06	1, 144.5	0.809
	Right	11.90 ± 0.52			
Eccentricity		-0.0012 ± 0.0002	73.5	1, 1007	<0.001
Eccentricity x Eye		-0.0001 ± 0.0003	0.03	1, 1013	0.87
Date sampled		0.0012 ± 0.0016	0.57	1, 26.38	0.46
Observer			10.4	3, 1040	<0.001

The dependent variable units were cells/1000mm^2;

†Eccentricity units were μm.

*Estimates: Sex and eye, LS means ± SE. Eccentricity and extraction date, partial β.

Eccentricity x Eye, the differential effect of the left eye compared to the right eye.

Table S6.8. F-tests of the effects of eye measures on cell densities, for total densities, each type of cone, and for the single cones together.

Cell type	Eye measure	F	df	p
Total	Axial length	0.30	1, 16.51	0.59
	Transverse length	0.07	1, 21.43	0.80
	Corneal diameter	0.74	1, 15.66	0.40
LWS	Axial length	0.28	1, 20.03	0.60
	Transverse length	0.43	1, 24.33	0.52
	Corneal diameter	0.63	1, 18.40	0.44
MWS	Axial length	0.08	1, 20.01	0.78
	Transverse length	0.46	1, 26.33	0.50
	Corneal diameter	0.10	1, 20.15	0.75
SWS	Axial length	0.02	1, 18.38	0.89
	Transverse length	0.09	1, 22.57	0.76
	Corneal diameter	0.63	1, 16.06	0.44
UVS	Axial length	0.00	1, 19.28	1.00
	Transverse length	1.13	1, 24.68	0.30
	Corneal diameter	0.03	1, 21.57	0.86
Double	Axial length	1.29	1, 12.23	0.28
	Transverse length	0.98	1, 16.00	0.34
	Corneal diameter	0.13	1, 13.26	0.72
Single	Axial length	0.04	1, 18.79	0.84
	Transverse length	0.40	1, 23.43	0.53
	Corneal diameter	0.75	1, 16.95	0.40
